# Transmission Sensitivities of Contact Ultrasonic Transducers and Their Applications

**DOI:** 10.3390/s21134396

**Published:** 2021-06-27

**Authors:** Kanji Ono, Hideo Cho, Hartmut Vallen, Robert T. M’Closkey

**Affiliations:** 1 Department of Materials Science and Engineering, University of California, Los Angeles, CA 90095, USA; 2 Department of Mechanical Engineering, Aoyama Gakuin University, Sagamihara 252-5258, Japan; cho@me.aoyama.ac.jp; 3 Vallen Systeme GmbH, 82515 Wolfratshausen, Germany; hartmut@vallen.de; 4 Department of Mechanical and Aerospace Engineering, University of California, Los Angeles, CA 90095, USA; rtm@seas.ucla.edu

**Keywords:** ultrasonic transducers, transmission sensitivities, calibration, acoustic emission, areal and multiple sensing methods, capacitive sensing, loading effect, receiving sensitivities, antiresonance

## Abstract

In all ultrasonic material evaluation methods, transducers and sensors play a key role of mechanoelectrical conversion. Their transduction characteristics must be known quantitatively in designing and implementing successful structural health monitoring (SHM) systems. Yet, their calibration and verification have lagged behind most other aspects of SHM system development. This study aims to extend recent advances in quantifying the transmission and receiving sensitivities to normally incident longitudinal waves of ultrasonic transducers and acoustic emission sensors. This paper covers extending the range of detection to lower frequencies, expanding to areal and multiple sensing methods and examining transducer loading effects. Using the refined transmission characteristics, the receiving sensitivities of transducers and sensors were reexamined under the conditions representing their actual usage. Results confirm that the interfacial wave transmission is governed by wave propagation theory and that the receiving sensitivity of resonant acoustic emission sensors peaks at antiresonance.

## 1. Introduction

The use of ultrasonic material evaluation methods started from Firestone’s work [1]. By the late 1950s, they had become an indispensable industrial tool as a part of nondestructive evaluation (NDE) methods. This was most prominent especially for the inspection of large turbine rotor steel forgings [2]. Applications of ultrasonic testing (UT) rapidly expanded in both engineering and medicine, primarily focusing on the detection of internal defects and irregularities [3,4]. Another ultrasonic method, acoustic emission (AE), emerged by the early 1960s and offered a valuable quality assurance tool for rocket motorcases, made of steels or fiber-reinforced composites [5]. AE was used to locate growing flaws globally by detecting ultrasonic waves emitted by active flaws using multiple receiving sensors. During the last 20 years, both approaches have become integral parts of structural health monitoring (SHM) and provided essential means of flaw characterization [6]. Medical ultrasonics cover broader physical phenomena than those in SHM applications [4], since ultrasonic intensity must be tightly controlled for patient’s safety in some medical applications. Still, the need to define the field strength distribution is common to both.

In all the areas of ultrasonic applications, transducers act as the interface between the mechanical and electrical parts of instrumentation. The conversion between mechanical and electrical signals usually relies on piezoelectric element(s), since other methods are based on electrostatic, electromagnetic and optical principles and are impractical from their cost, size and/or sensitivity. Mason [7] introduced the equivalent circuit analysis to model the electrical behavior of a piezoelectric transducer. It has been refined by adding a delay line to account for wave propagation [8] and by using more elaborate computer modeling [9]. Electromechanical characterization of an immersible transducer (or a hydrophone) relied first on a reciprocity method, followed by the use of optical interferometry. Although the reciprocity method is applicable only to reciprocal transducers, the sound field of a test tank can be characterized, allowing the use of a substitution method. This procedure was superseded by the use of a pellicle and a laser interferometer [10]. 

Today, interferometric methods can calibrate a hydrophone up to 100 MHz at two national standards laboratories [11,12]. For ultrasonic works in SHM and industrial NDE, low MHz frequencies are typically used, and many applications utilize contact transducers because of practicality and cost. For UT and AE transducers, their calibration utilizes optical interferometry, capacitive sensing and substitution methods [13,14,15,16,17,18,19,20,21,22]. The transmission sensitivity (displacement or velocity per unit input voltage) can be readily determined by pulse excitation plus laser interferometry, but was not reported for broadband UT transducers until recently [21,22]. The receiving sensitivity (output voltage per unit displacement or velocity) of five AE sensors was first obtained by Matsuda et al. [18,19] using optical interferometry plus direct contact method, joining a transmitter and receiver face-to-face. More results for 27 AE sensors have been published [23].

AE sensors are used by coupling them to the surfaces of structures under surveillance. Eitzen and Breckenridge [24] demonstrated large effects of the acoustic impedance (Z = *ρ*v = (mass density) × (wave velocity)) of the surface material on the receiving sensitivities of two sensors. For the reception of Rayleigh waves on aluminum (Al) and polymethyl methacrylate (PMMA), the receiving sensitivities decreased by at least 8 to 25 dB in comparison to the corresponding values on steel. Sause and Hamstad [25] recently showed similar effects, smaller in magnitude down to no effect, for both normally incident bulk waves and Rayleigh waves through modeling. These effects come from the transmission of acoustic waves through an interface, where the acoustic impedance changes abruptly from Z_1_ to Z_2_. Displacement ratio, ξ_2_/ξ_1_, is given by [26] as

ξ_2_/ξ_1_ = 2Z_1_/(Z_1_ + Z_2_). (1)

Displacement ratio from Equation (1) is independent of frequency, f, but the effects reported in [24,25] varied widely with frequency. For the cases of Rayleigh waves, aperture effect caused frequency-dependent sensitivity changes [24]. AE sensors and UT transducers usually have thin alumina face plates. Average Z value of three similar alumina plates (0.63-mm thick, 25-mm square or round disks: Materials Research Corp, Orangeburg, NY, USA or All Electronics, Van Nuys, CA, USA) was found to be 32.9 Mrayl. This is smaller than Z = 37–39 Mrayl for near-theoretical density alumina with *ρ* = 3.98 [3] as their *ρ* values were 3.74 to 3.84 Mg/m^3^, averaging at 3.80 Mg/m^3^ (95.7% dense). Taking Z values of steel, Al and PMMA as 46.5, 17 and 3.2 Mrayl [3], displacement ratios for Al and PMMA were 4.7 and 16.4 dB lower than that for steel, differing significantly from the reported behavior [25]. Since this aspect critically affects the receiving sensitivities of AE sensors, a section is devoted to this subject. 

While the basic method of characterizing the transmission sensitivity of a UT transducer is straight-forward, some issues need further consideration. In particular, it is necessary to extend the frequency range below 20 kHz (the low frequency limit in references [21,22,23]) and to consider effects of point vs. area sensing as well as transducer loading effects. The latter aspects are important elements of international endeavor at the International Organization for Standardization (ISO). At ISO committee TC135/SC9, Project 24543 is in progress to address the verification of the receiving sensitivity of AE sensors in a face-to-face set-up using an ultrasonic transducer as transmitter. Capacitive and small aperture sensors are also utilized for comparison with laser interferometry. The present study is aimed at resolving these issues with thorough evaluation of various related approaches.

## 2. Experimental Procedures

Transducers evaluated in this study are listed in Table 1 with their nominal center frequencies and aperture (element) sizes. All were the non-focusing type. All eight Olympus transducers (Olympus NDT Instruments, Waltham, MA, USA) were of the damped broadband type except for V195, which seems to have an internal tuning inductor. The broadband type also included NDT C16 (NDT Systems, Huntington Beach, CA, USA). AE sensors included R15 and R15a (Physical Acoustics, Princeton Junction, NJ, USA), which are resonant AE sensors, and F30a and WD (Physical Acoustics) and KRNBB (KRN Services, Richland, WA, USA) that are of the broadband type. 

Laser interferometers were used as the primary calibration devices. For frequencies of 20 kHz to ~15 MHz, Thales SH-140 interferometer (Thales Laser, Orsay, France) was used as previously reported in [21,22]. In these tests, a transducer was excited by a home-built pulser or by continuous sine waves through a power amplifier (HSA4011, NF Corp., Yokohama, Japan). The continuous excitation was only used for V189. For frequencies of 0.1 to 100 kHz, a laser vibrometer (OFV3001/OFV512, Polytec, Waldbronn, Germany) was used for three Olympus transducers (V101, V104 and V189) in combination with a dynamic signal analyzer (SR785, Stanford Research Systems, Sunnyvale, CA, USA) and a power amplifier (KC1000-70, Kinetic Ceramics, Ambler, PA, USA). A chirp signal stimulus was applied to a transducer. Random signal excitation was also used down to 0.1 kHz, producing nearly identical results at overlapping frequencies. In the laser interferometry calibration using pulse excitation, the output displacement spectrum was divided by the input pulse spectrum, resulting in the transmission sensitivity, T_v_, expressed in dB in reference to 0 dB at 1 nm/V. Noesis software (ver. 5.8, Enviroacoustics, Athens, Greece) was used for fast Fourier transform (FFT), while most signal acquisition used Picoscope (5242D, Pico Technology, St Neots, UK). In conducting FFT, spectral resolution was increased four times (from 1.9 to 0.48 kHz) compared to earlier analyses [21,22].

**Figure 1:**
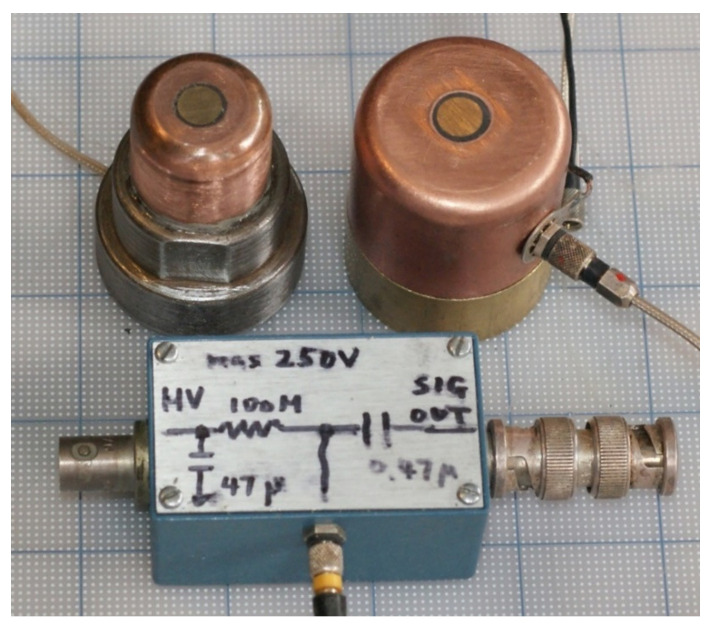
Capacitive displacement sensors (CS-16, **left** and CS-30, **right**) and a junction box.

**Figure 2:**
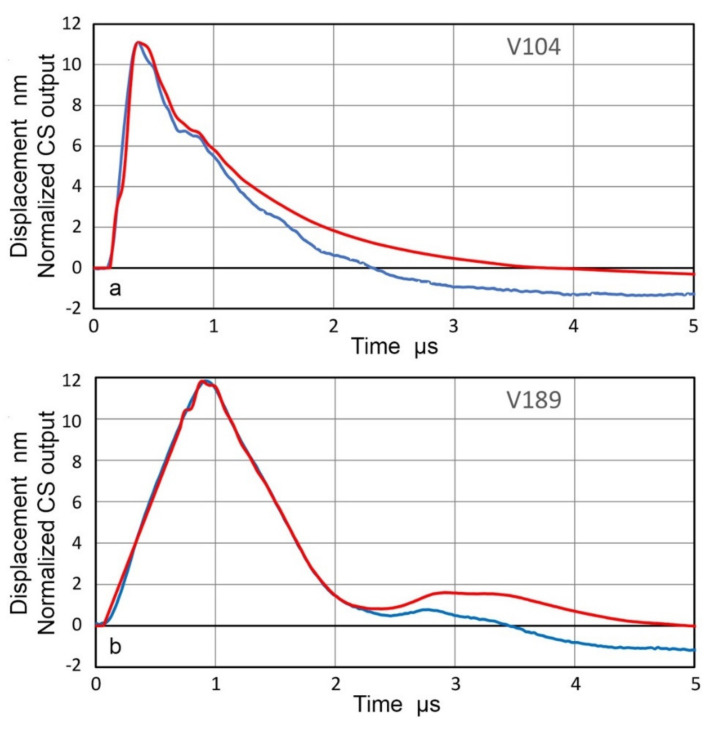
Matching waveforms of LI-based displacement (blue curve) and normalized CS output (red curve). (**a**) V104 transducer. (**b**) V189 transducer. Short pulse excitation was used.

A capacitive displacement sensor was built using a brass center electrode of 7.63-mm diameter and 25-mm diameter copper casing. Ground contact area was 16 mm in diameter. The center electrode was electropolished using a phosphoric acid electrolyte, producing the sensor capacitance, C_o_ = 25.1 ± 3.7 pF, on a flat conductor or on a transducer with gold conductive coating or burnished foil. This corresponds to an air gap, g = 16.4 ± 2.4 µm. At the bias voltage of V_o_ = 150.5 V and stray capacitance, C_s_ = 51.4 pF, its displacement sensitivity (S_cs_) was calculated as:

S_cs_ = (V_o_/(C_o_ + C_s_))·dC_o_/dg = 33.52 log C_o_ − 97.23 (2)

(in dB: referenced to 0 dB at 1 V/nm). Another capacitive displacement sensor was built using a 7.90-mm diameter electrode and a larger casing of 38-mm diameter with 30-mm diameter ground contact. These are shown in Figure 1. These two sensors are designated as CS-16 and CS-30, referring to the ground contact area of 16-mm or 30-mm diameter. The larger casing size was needed for larger transducers at lower frequencies, as will be discussed. The displacement sensitivity for a given experimental set-up was calibrated by comparing the peak output voltage to the peak displacement output determined by laser interferometry for the same transducer, excited by the same driving pulse. Two examples are shown in Figure 2. Here, the capacitive sensor output was adjusted to match at the peak displacement output in order to illustrate good correspondence of the two waveforms through the initial impulse. 

**Figure 3:**
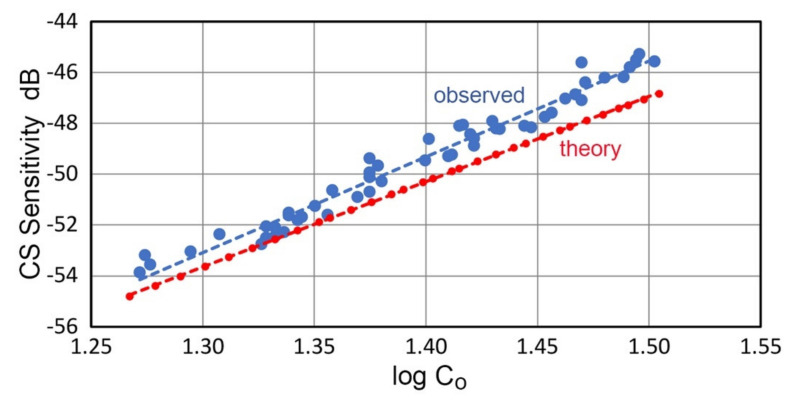
Observed displacement sensitivities of CS-16 sensor calibrated using LI-based peak displacement (**blue dots**). Theoretical displacement sensitivities were calculated from C_o_ values (**red dots**).

The calculated and observed displacement sensitivity (S_cs_) values are plotted against the logarithm of C_o_ values (in pF) in Figure 3. The average observed sensitivity of the CS-16 sensor was S_cs_ = −49.5 ± 2.4 dB in reference to 0 dB at 1 V/nm (for the sample size of 55). The average sensitivity of CS-30 sensor was −65.7 dB since a larger air gap (36~48 µm) was needed due to its larger ground contact area. Observed S_cs_ values of CS-16 increased with C_o_ and varied as:

S_cs_ = 37.7 log C_o_ − 102.0 (3)

and were higher than the calculated S_cs_ values for the same C_o_ by ∆S_cs_ = 0.97 ± 0.29 dB on average. The slope of the observed S_cs_ was 1.88, which was 0.21 higher than that of the calculated S_cs_. Both plots follow straight lines, and data scatter was approximately ±0.5 dB for the observed S_cs_. The observed S_cs_ value for a given test set-up was used for further analysis since it was calibrated with laser interferometry result. Note that both laser interferometry and capacitive displacement sensing are non-contact methods, but the latter gives the average sensitivity over 45.7 mm^2^ (or 49.0 mm^2^ for CS-30) area, producing differences in the sensitivity spectra, especially below 50 kHz. The spectral results will be discussed in a later section. For this part, capacitance was measured using LCR-45 capacitance meter from Peak Atlas (Buxton, UK). Its nominal precision is ±0.2 pF. The difference between observed and calculated S_cs_ values (currently 0.97 dB) may change by improving the accuracy of the capacitance measurements, especially using a calibrated capacitance standard. Note that observed S_cs_ values together with the calibrated Olympus transducers require no capacitance measurements since these rely on the laser interferometric data. The laser data is available for all the transducers used in this work except KRN sensor.

Using the transmission sensitivity, T_v_, of Olympus V101, V104 and V189 transducers, the low frequency T_v_ spectra (for 1 to 100 kHz) of other transducers were evaluated by an indirect method. The receiving sensitivities, R_v_, of other transducers were obtained by spectral division with direct contact testing, as previously reported [21,22]. R_v_ spectrum thus obtained can be used to determine T_v_ of another transducer. While this indirect method introduces additional error, T_v_ can be estimated when laser interferometry is unavailable. The direct contact testing will also be used to evaluate effects of transducer loading. This examination is always needed, but especially when a high sensitivity AE sensor is used as a transmitter. In this case, transmission behavior is strongly affected by coupling it directly with another sensor, making resonant AE sensors to be inappropriate as transmitters for face-to-face calibration.

In most of previous transmission sensitivity tests, a short pulse excitation was used. While this was adequate for the frequency range of interest, 20 kHz to 15 MHz, it was necessary to consider other excitation schemes at lower frequencies. Uses of continuous sine waves, chirp waveforms, and longer pulse waveforms were examined with respect to T_v_ spectra. For these tests, the arbitrary waveform generator function of Picoscope 5242D and a power amplifier (HP467A, Hewlett Packard, Palo Alto, CA, USA) were used. We also used the same pulse generator as before, but removed the 50-Ω shunt resistor or increased the shunt to 10 kΩ. These steps increased the low frequency contents of the excitation pulses.

## 3. Results and Discussion

### 3.1. Low-Frequency Transmission Sensitivity

**Figure 4:**
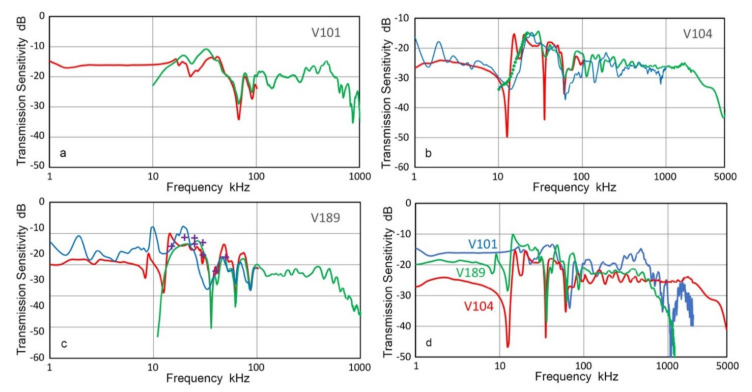
Transmission sensitivities of three transducers from laser interferometry. Red curves are from Polytec vibrometer and green curves from Thales interferometer. Blue curves in (**b**) and (**c**) are T_v_ spectra from indirect method (see text). Also shown in purple + symbols are Thales data using sine wave excitation. (**a**) V101. (**b**) V104. (**c**) V189. (**d**) All three shown together.

The transmission sensitivities, T_v_, of three Olympus transducers (V101, V104 and V189) are shown in Figure 4. In Figure 4a–c, T_v_-spectra of both low-frequency (under 100 kHz, using chirp) and high-frequency (above 20 kHz, using pulse) tests are given in red and green curves. The transition from the low-frequency spectra to high-frequency spectra matches well. Since the low-frequency data was obtained at a higher resolution using chirp signals, the high-frequency data were used above 80 to 100 kHz, selecting to make a smooth transition. Three combined spectra are shown in Figure 4d. In the case of V189 transducer, sine wave test data was available between 20 and 50 kHz and plotted in purple + symbols in Figure 4c. These matched well with both chirp and pulse interferometric results. The sine wave data was obtained using the same SH-140 interferometer as the pulse data.

The above results showed that the two interferometers used provide consistent displacement data. Although SH-140 interferometer includes a high-pass filter with 20-kHz cut-off frequency, T_v_ results agree with the low-frequency Polytec interferometer down to 15 kHz. In all three transducers, T_v_-spectra exhibited approximately flat responses over the entire frequency range below the respective center frequencies, but with lower frequency peaks and dips. This general trend is consistent with the expected behavior of damped piezoelectric elements. However, T_v_ values decreased sharply above the nominal center frequency of each transducer. Additionally, a series of broad peaks and sharp dips were observed from 10 kHz to 100 kHz. The depth of the dip was deeper with the chirp excitation, but the frequencies of the dips were similar between the two excitation methods. Some of the dip frequencies at 30–100 kHz appear to arise from the radial resonances of the transducer elements of 25 or 38 mm in diameter, but some lower frequency dips are difficult to attribute to radial resonance. Broad peaks are also difficult to attribute to resonance behavior or to possible causes. Further study is needed to account for these peaks and dips. 

In order to examine the origin of prominent sharp dips in T_v_ spectrum of V104 transducer obtained using Polytec vibrometer, additional tests were conducted using indirect method discussed next and resultant T_v_ is shown by blue curve in Figure 4b. This indirect T_v_ spectrum matched well to two previous spectra from laser interferometry except at two sharp dips observed at 12.4 and 35.3 kHz in the Polytec spectrum. In the indirect T_v_, the two dips vanished, while a dip near 60 kHz was present in all three spectra. The two lower dip frequencies are approximately in the ratio 1:3, indicating a possible antiresonance, which was suppressed by the contact to a receiver (KRNBB sensor, calibrated using V101 T_v_ data in Figure 4a) in the indirect method. This suggests the presence of a subsurface flaw in this unit of V104, which requires contact pressure for proper transduction. The indirect method was also used to obtain T_v_ spectrum of V189 transducer, shown in Figure 4c. Two lower frequency dips were absent, while the general trends were similar among the three spectra. Again, contact pressure suppressed the unwanted dips in T_v_. These findings showed the needs for properly accounting for the front-face loading effects, but a suitable method is yet to be developed.

**Figure 5:**
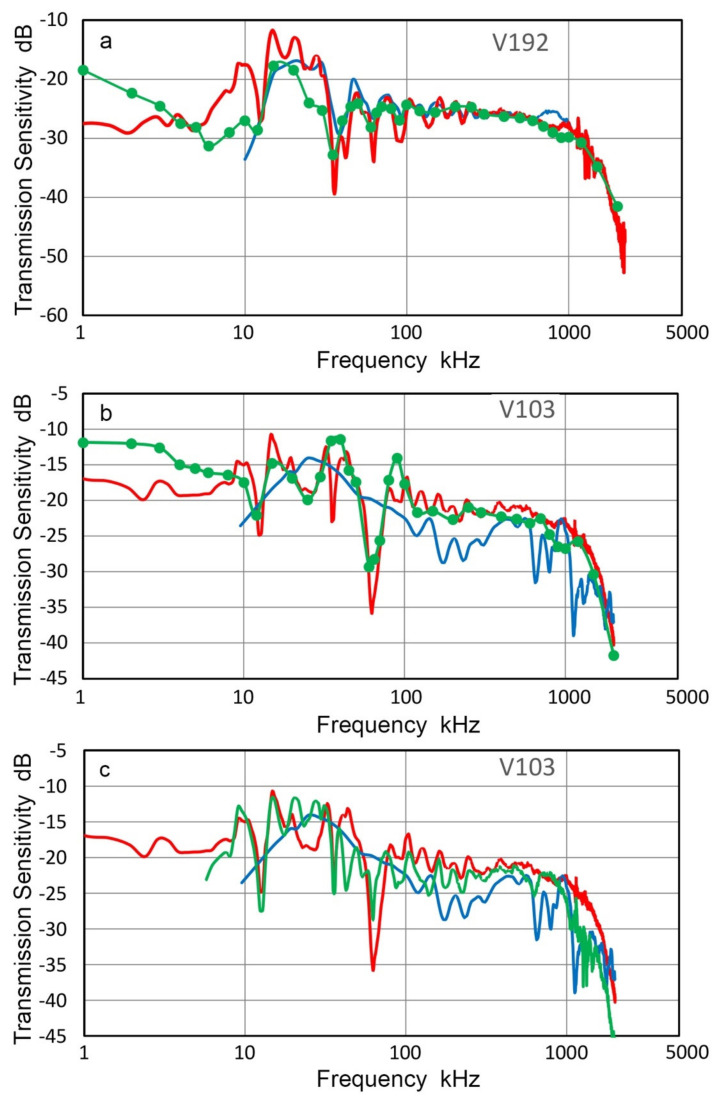
Transmission sensitivities of two transducers. (**a**) V192. (**b**,**c**) V103, using indirect method. Blue curves are from Thales interferometer for comparison, red and green dotted curves are from the indirect method. Three receivers, V101, V104 and V189 were used and spectral results averaged. Red curve used short pulse excitation while green dotted curve was with sine wave excitation. For green curve in (**c**), KRNBB receiver was used on six positions and spectral results averaged.

Two transducers, Olympus V192 and V103 (both with 1-MHz center frequency), were tested for their T_v_-spectra using an indirect method; that is, the R_v_-spectra of V101, V104 and V189 were obtained and these were used to determine the T_v_-spectra of V192 and V103. Both short-pulse and sine wave excitation methods were used. Figure 5 shows the results. Red curves are for T_v_-spectra with pulse excitation, green dots are for sine wave excitation at discrete frequencies (connected by interpolated curves) and blue curves are previously reported T_v_-spectrum from laser interferometry at frequencies above 10 kHz. All the results are the average spectra from three tests, which used three R_v_-spectra of V101, V104 and V189, respectively. Three T_v_ spectra of V192 showed good agreement above 12 kHz except at 25–30 kHz (Figure 5a). Direct laser spectral result given in blue curve matched well with the new T_v_ data, especially with the pulse data (red). However, deviations between short-pulse and sine wave excitation test results were large below 10 kHz, reaching nearly 10 dB. The corresponding T_v_ results for V103 (Figure 5b) showed good agreement between short-pulse and sine wave excitation test results, but these deviated from the direct laser spectrum below 400 kHz. T_v_ values were similar in magnitude, but the spectral variation of the direct laser data was different from the indirect test results. This is likely due to the single point sensing, as discussed in the next section, whereas the indirect method averages the sensor response of the entire sensor face. Another series of tests were conducted using a small aperture receiver, KRNBB. Six positions are two each at the center, ±2.5 mm and ±5.0 mm off-center. 

In Figure 5c, spectral results were averaged and compared to those from the direct (blue) and indirect (red) methods given in Figure 5b. In this case, the multi-point sensing result with the KRNBB sensor matched with the indirect spectral result. Note that simple and area-weighted averages (to be introduced in Section 3.3) were almost identical with the average difference of 0.03 dB. Consequently, the use of multi-point sensing with laser interferometer is expected to significantly reduce the deviation observed in Figure 5b. T_v_ result for V104 obtained using indirect method (cf. Figure 4b) was compared to laser interferometric spectra. This test consisted of ten T_v_ determination on V104 surface using KRNBB sensor as above. The positions used were from the center to ±10 mm radial distances in 2.5-mm steps. The overall agreement was good between the averaged indirect and laser-based T_v_ spectra. The cause of spectral dips observed on one of the T_v_ spectra was traced to a resonance of the V104 unit under unloaded surface condition. 

In addition to the indirect method discussed above, a tri-transducer method (TTM) was also used in earlier studies [21,22]. This also relied on face-to-face tests, and TTM implicitly assumed that direct contact introduces no significant changes in the transmission and receiving sensitivities. For broadband damped UT transducers, this assumption was deemed mostly valid as T_v_ results from TTM agreed with those of direct laser interferometric test results. 

In this section, T_v_-spectra determination was extended to lower frequencies using a laser vibrometer. Some spurious dips and peaks were noted in a V104 unit tested. These vanished when indirect method with a contact receiver was used. The indirect method evaluated can also be used to estimate the transmission characteristics of damped UT transducers when laser interferometers are unavailable. 

### 3.2. Positional and Areal Transmission Sensitivities 

**Figure 6:**
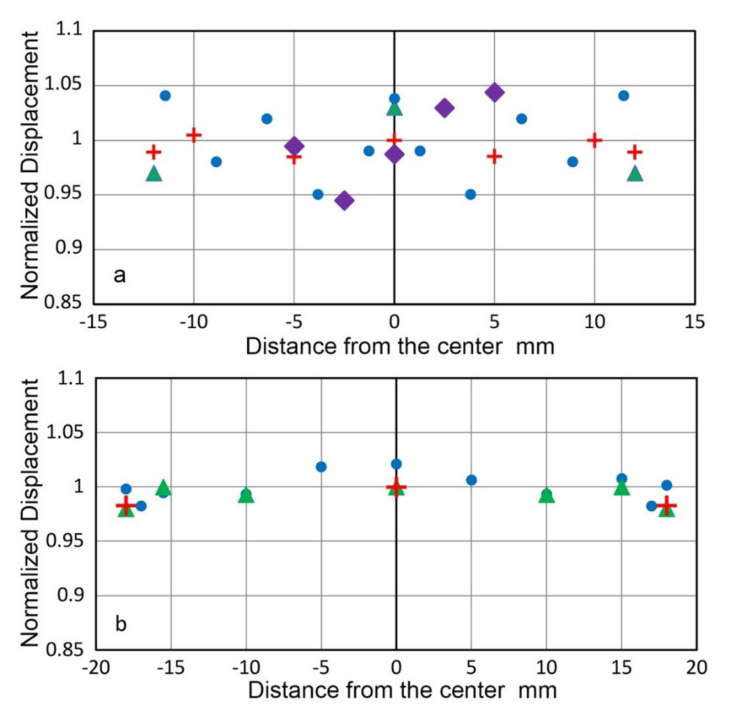
Variation of peak surface displacement obtained by laser interferometry or by a small receiver vs. distance from the center position. (**a**) Three laser tests on two V104 (blue, red and green, see text). KRNBB sensor test on V103 shown in purple diamond. (**b**) Green triangles and red + symbols were LI-based on V192 and V195, respectively, while blue dots were from KRNBB sensor test on V192.

In previous laser interferometric studies of transmission sensitivities, T_v_, a few additional spots were chosen to determine displacement spectra away from the center position. Noting that the peak amplitude is within ±0.3 dB for Olympus V104, V192 and V195, it was assumed that the central T_v_ spectrum adequately represents the transducer behavior. Observed positional dependence of peak displacement amplitude is shown in Figure 6. In Figure 6a, blue dots are for V104, tested using a Picoscale scanning vibrometer with F03 sensor head (SmarAct, Oldenburg, Germany). Two other V104 sets (green triangles and red + symbols) used Thales SH-140, as noted previously. Amplitude data for V192 (green triangles) and for V195 (red + symbols) in Figure 6b used SH-140. Another approach was to use a small aperture AE sensor (KRNBB-PCP, KRN Services, Richland, WA, USA) to actually measure the distribution of peak displacement outputs and T_v_ spectra, as shown in Figure 6a (purple diamond) for Olympus V103 and in Figure 6b (blue dots, data from [27]) for Olympus V192. Olympus V103 transducer exhibited the peak amplitude variation of ±0.34 dB. Its average T_v_ spectrum was plotted in Figure 5c above and the standard deviation of the spectra was 2.37 dB over 6 kHz to 2 MHz. Olympus V192 transducer exhibited the peak amplitude variation of ±0.26 dB. For this case, T_v_ spectrum changes were <2 dB [27]. 

**Figure 7:**
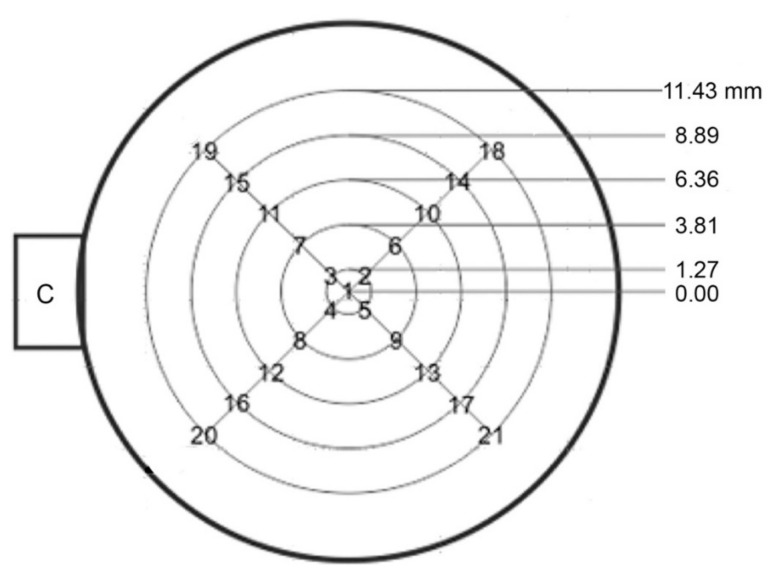
Laser measurement positions on V104. C is the location of a connector. A total of 22 displacement measurements were conducted. These were at positions 2 to 21 on five rings and two tests at the center (position 1). Ring radii are shown.

Recent introduction of scanning laser vibrometers has allowed detailed characterization of T_v_ spectra over the entire surface area of a transducer. Results on a new Olympus V104 transducer are presented in this section. For this work, conducted at Vallen Systeme GmbH (Wolfratshausen, Germany), a Picoscale scanning vibrometer with F03 sensor head was used. Its programmable scanning capability gives 0.01-mm precision in sensor head positioning. A total of 20 positions were selected on five coaxial rings plus the center (measured twice), as shown in Figure 7. At each position, decoded signals were sampled at 10 MHz and averaged 10,000 times, while V104 was excited by 1-MHz cosine pulse of 18 V peak-to-peak amplitude. 

**Figure 8:**
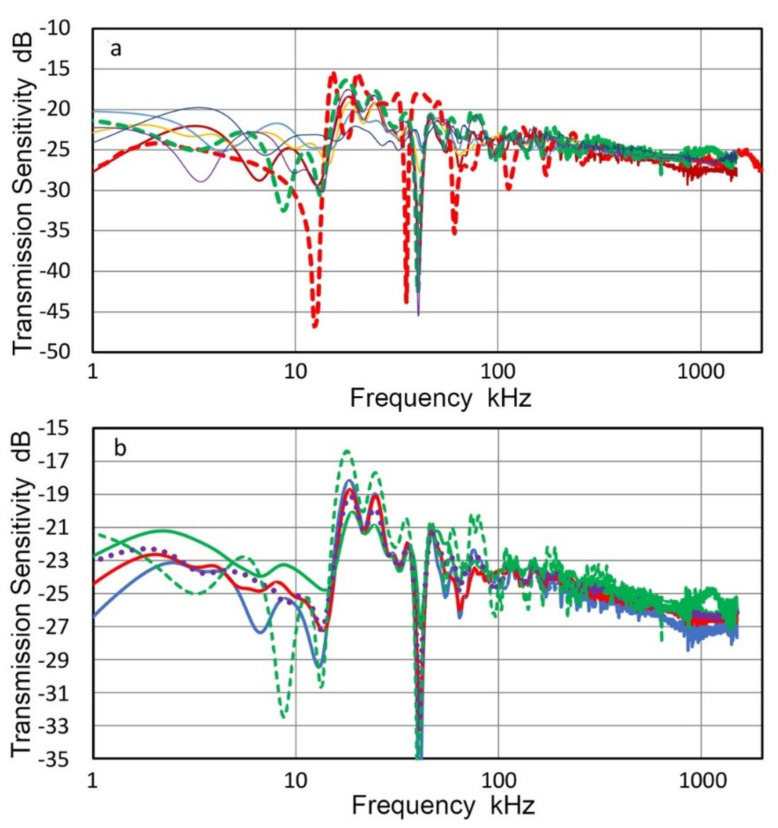
LI-based transmission sensitivities of V104. (**a**) Green dash curve at the center. Purple, brown, yellow, blue and black curves are for Areas 1 through 5, respectively. The red dash curve is the central T_v_ for another V104 from Figure 4b for comparison. (**b**) Green dash curve at the center as in Figure 8a. The blue, red and green curves are for weighted areal sensitivities of Areas 2, 3 and 5 (10.2 mm, 15.2 mm and 25.4 mm diameter), respectively. The purple dot curve is the simple average of Rings 0–5 transmission sensitivities.

From the displacement data of 22 measurements, T_v_ spectra were calculated for each position. These were averaged for the center (two tests) and for five rings (four tests each). Results are given in Figure 8a with color-coded curves. The center (Ring 0) spectrum is in green dash curve, while five other ring data are in purple (Ring 1), dark red (Ring 2), orange (Ring 3), blue (Ring 4) and dark blue (Ring 5), respectively. Ring radii are 0, 1.27, 3.81, 6.35, 8.89 and 11.43 mm, as shown in Figure 7. Here, we define concentric rings, Area 0 to Area 5, which correspond to the six T_v_ spectra. Each area is bounded by the mid-positions between rings. That is, Area 0 is a circle of radius r = 0.635 mm and Area 1 is a ring with r = 2.54 mm. For Areas 2, 3, 4 and 5, r = 5.09, 7.63, 10.16 and 12.7 mm, respectively, limited by the aperture radius of V104. 

These six T_v_ spectral curves are in good agreement from 20 kHz to 1.5 MHz, which is the intended frequency range for the Vallen study. In addition, the corresponding spectrum of V104 is shown for comparison in Figure 4b (red dash curve). This curve matched well with the six T_v_ spectral curves, but had a few extra dips in the T_v_ spectrum and only one of them had a corresponding dip. When a receiver is in contact with a transmitter, displacement output transferred to the receiver also depends on the area. Thus, outer ring areas become more dominant and this effect must be included in receiver characterization. The area fractions of contributing rings are given in Table 2.

The dominance of the outer rings is clearly indicated in Table 2, implying the importance of determining T_v_ spectra at multiple positions. T_v_ spectra incorporating variable contributions of different ring areas were calculated for the three cases, Areas 2, 3 and 5 in Table 2. These are shown in Figure 8b with blue curve for the outer diameters of 10.2 mm, red curve for 15.3 mm diameter and green curve for 25.4 mm diameter. The T_v_ spectrum at the center (blue dash curve) and the unweighted average spectrum of center to Ring 5 T_v_ spectra (purple dotted curve) are also shown. Deviations from the central spectrum to areal averaged spectra persist over the entire frequency range, reaching more than 3 dB. The use of areal averaging clearly improves the accuracy of receiver sensitivity determination, although it requires advanced instrumentation and additional signal processing steps. Above 15 kHz, all the curves are in good agreement within ±1.5 dB, except at peaks and especially at the big dip at 41 kHz. Surprisingly, the simple averaging also produced a good match above 15 kHz and can be used as a good approximation to the weighted average spectra. Of course, this does not lessen the desirability of defining T_v_ spectra at multiple positions and properly partitioning the outcome.

In the above V104 tests at Vallen Systeme GmbH, the observed positional dependence of peak displacement amplitude was also small at ±0.4 dB as shown in Figure 6a (blue dots). Yet, T_v_ spectral variations reached 3 dB. This indicates that the flatness of peak displacement values cannot be used to justify the use of single-point T_v_ spectrum determination unless a few dB error can be tolerated. In another set of positional dependence tests of V103, given in the previous section, the displacement amplitude variation was 0.37 dB and T_v_ spectral variations of 2.4 dB were found. These values are consistent with those for V104, although V103 tests utilized a small aperture receiver in place of laser vibrometer. Thus, the method using a small aperture receiver can be useful at a fraction of laser testing cost.

Results of this section indicate the desirability of using multiple point sensing in determining the T_v_ spectrum of a transducer. It is definitely superior to single point sensing method, which can still characterize the transmission sensitivity reasonably well. A scanning laser interferometer provides non-contact precision positioning and reliable displacement measurements, but a small aperture receiver can also map position-dependent T_v_ spectra adequately, especially when the receiver is calibrated using a laser interferometer. The positional precision of this method can be enhanced by using a suitable scanning device.

### 3.3. Capacitive Sensing of Transmission Sensitivities

Another non-contact capacitive sensing (CS) method is used to obtain transmission sensitivities of transmitters. Two capacitance sensors (CS-16 and CS-30) were built and used with five broadband transducers, Olympus V189, V101, V104, V192 and V107. These have electrodes of 7.6 and 7.9-mm diameter and provide areal sensing in contrast to point sensing of laser interferometry. Short pulse excitation was used to calibrate the capacitance sensor for each set-up by comparing the displacement waveform from its laser interferometric test with the output of the capacitance sensor. Other excitation methods (longer decay pulse, chirp and discrete sine wave) were also used while keeping the sensor set-up steady under 4 N static force. 

**Figure 9:**
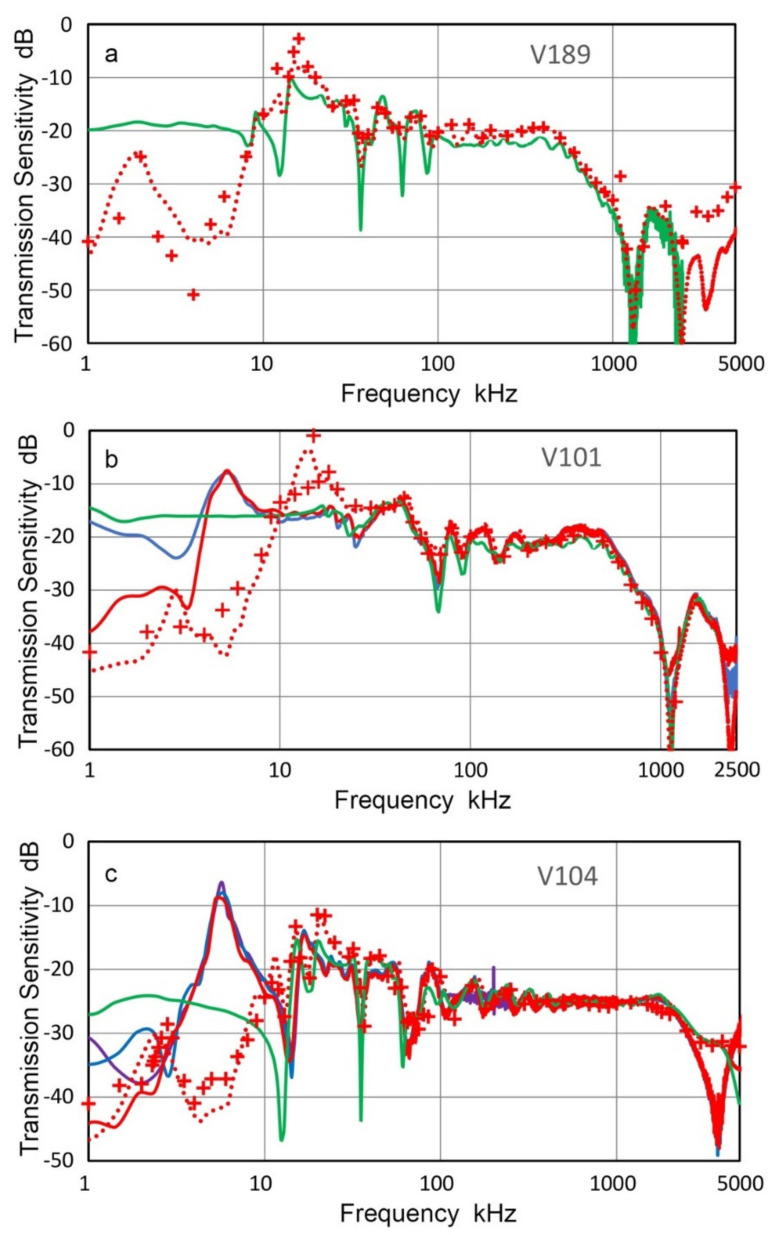
Comparison of LI-based (green curves) and CS-based transmission sensitivities. Red dot curves: CS-16 using short pulse excitation of a transmitter. Red + symbols: CS-16 using sine wave excitation. Red curves: CS-30 using long pulse excitation. Blue curve: CS-30 using short pulse excitation. Purple curve: CS-30 using chirp excitation. (**a**) V189. (**b**) V101. (**c**) V104.

T_v_ spectra of V189 (0.5 MHz, 38 mm) obtained using capacitive sensing and laser interferometry (LI) are plotted in Figure 9a. T_v_ spectrum with a capacitance sensor was the average of 10 tests using CS-16 (shown in red dot curve). For these CS tests, short pulse excitation was used. LI-based T_v_ spectrum (in green curve) combined two tests that used Polytec (below 100 kHz) and Thales (above 100 kHz) interferometers. The CS and LI spectra matched well between 25 kHz and 2.5 MHz, except CS data was ~2 dB higher at 100–400 kHz and large deviations were observed at spectral dips of the LI spectrum. The LI-based T_v_ spectrum became noisy above 2.5 MHz, but the CS-based T_v_ spectrum was recorded to 5 MHz without added noise. 

The red + symbols show discrete T_v_ values for CS testing that used sine wave trains for V189 excitation. Since the output was measured after initial transient, the discrete spectra represent the steady state responses. As shown in Figure 9a, the two CS test result agreed well to 2.5 MHz, but the discrete values became higher above 2.5 MHz. A better interferometer is needed to verify the performance of capacitance sensors at higher frequencies. At frequencies below 25 kHz, the T_v_ values of the capacitance sensor showed large fluctuations, with possible resonance peaks at 15 and 1.9 kHz. Since a peak exists at 14.3 kHz in the LI-based T_v_ spectrum, the 15-kHz peak may be of the same origin. However, it is found in other tests as well, and is likely from sensor resonance. The 1.9-kHz peak appears to originate from capacitance sensor design. Similar low-frequency resonance peaks were reported by Kim et al. [28] and capacitive sensing at the low-frequency region requires further study, requiring a more elaborate design as was used in [16] or commercial sensors. 

T_v_ spectra of V101 (0.5 MHz, 25.4 mm) by CS and LI tests are shown in Figure 9b. LI-based T_v_ spectrum is again in green curve, while three CS-based T_v_ spectra are shown plus the discrete spectrum. In this case, two capacitance sensors (CS-16 and CS-30) were used to compare effects of the size of ground contact. T_v_ spectra using CS-16 are in red dot curve and red + symbols, the latter for sine wave excitation. T_v_ values with CS-16 (ten repeat tests, averaged) matched with the LI-based spectrum well from 30 kHz to 2 MHz. Both of them exhibited peaks at or near 15 kHz and at ~3 kHz, as with V189 (Figure 9a). With a large size CS-30 (three repeat tests, averaged), T_v_ spectra matched with the LI-based spectrum above 8 kHz. Two excitation methods were used: short pulse (in blue curve) and long pulse with 1-ms decay time (in red curve), but no differences between the two curves were found above 4 kHz. A peak was observed at 5 kHz for both CS-30 spectra, indicating that a larger ground contact preserves their spectrum fidelity down to 8 kHz. When CS-16 is placed on a transmitter of 25-mm diameter, the entire base plane of CS-16 is subjected to the motion of the transmitter, causing a resonance at 15 kHz and reducing the sensitivity of CS-16 below this resonance. This effect diminishes at higher frequencies, above 30 kHz in the case of CS-16. Even though CS-30 has ground contact extending to the edge of the V101 transmitter, the same effect exists below 8 kHz. 

Results of the transmission sensitivities of V104 transducer (2.25 MHz, 25.4 mm) are shown in Figure 9c. Color coding of T_v_ spectra follows that of Figure 9b. In addition, a purple curve represents T_v_ spectrum for the case of using CS-30 sensor using a chirp excitation of 1 to 100 kHz. For the chirp excitation, T_v_ values were obtained up to 200 kHz and plotted. All three T_v_ spectra with CS-30 (red, blue and purple curves) were almost identical with one another down to 3 kHz and matched with the LI-based spectrum (green curve) from 13 kHz to 2.5 MHz. Two T_v_ spectra with CS-16 (red dot and red +) were close to each other and matched with the LI-based spectrum (green curve) from 13 kHz to 4.5 MHz. Matching was poorer at around 20 kHz for CS-16 case, however. Low frequency resonances for CS-16 and CS-30 were at 2.5 and 5.7 kHz, respectively.

**Figure 10:**
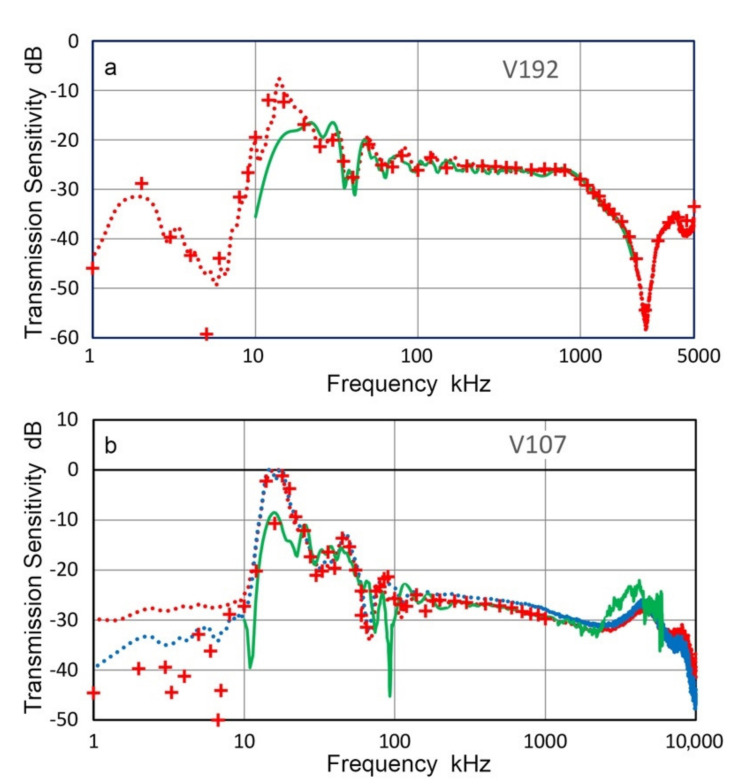
Comparison of LI-based (green curves) and CS-based transmission sensitivities. Red dot curves: CS-16 using short pulse excitation of a transmitter. Red + symbols: CS-16 using sine wave excitation. Blue dot curve: CS-16 using long pulse excitation. (**a**). V192. (**b**). V107.

The T_v_ spectra of V192 (1 MHz, 38 mm) by CS and LI tests are shown in Figure 10a. CS-16 sensor was used with short pulse and sine wave excitation and results plotted in red dot curve and red + symbols. LI-based spectrum only used Thales interferometer. Three spectra matched well from 20 kHz to 2.2 MHz. Two CS-16 T_v_ spectral results continued to show good match to 5 MHz, even though the laser results became too noisy to show. Low frequency resonances for CS-16 were again found at 14 and 2 kHz, similar to other cases discussed above.

The transmission sensitivities of V107 transducer (5 MHz, 25.4 mm) are shown in Figure 10b. Again, LI-based spectrum only used Thales interferometer, giving T_v_ up to 6 MHz (green curve). CS-16 sensor was used with short pulse, long pulse and sine wave excitation and results plotted in red dot curve, blue dot curve and red + symbols. These CS-based data matched one another well from 10 kHz to 1 MHz. Two dotted curves showed the same trend but with slight differences (<2 dB) in magnitude to 10 MHz. Matching between LI-based and CS-based T_v_ values were good from 25 kHz to 2.5 MHz. Above 2.5 MHz, LI-based T_v_ showed a peak at 4.3 MHz and exceeded CS counterpart to 6 MHz. The high frequency region needs further evaluation by conducting both CS and LI tests. 15-kHz resonance in CS result is again present, although it may be partly from T_v_ behavior.

Results of T_v_ spectral determination using capacitive sensors presented in this section can be summarized as follows:

(a) Capacitive sensors can measure area-averaged surface displacements above their low-frequency resonance at 10 to 30 kHz. The sensors used had the displacement sensitivity of −45 to −65 dB in reference to 0 dB at 1 V/nm.
(b) The results are quantitatively comparable to laser interferometry and have less spectral fluctuations than point-sensing LI-based spectra.
(c) The high frequency sensing limit appears to be about 5 MHz for capacitive sensors of simple design used here, but the limit should be extendable with attention to proper electromechanical design.

### 3.4. Transmission Sensitivities of Resonant Transducers

**Figure 11:**
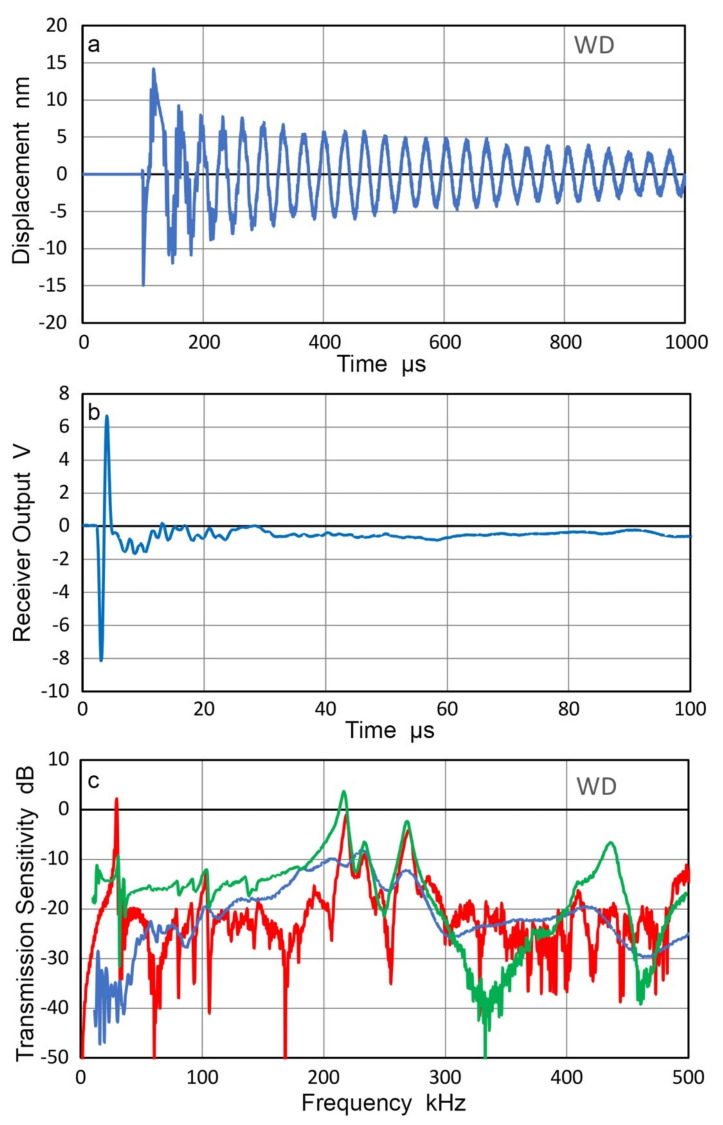
(**a**) LI-based displacement (in nm) of step-pulse excited WD sensor. (**b**) V103 receiver output (in V) in contact with short pulse excited WD. (**c**) Transmission sensitivities of WD from LI-based detection (red curve); from CS-16 sensor (green curve); from indirect method using V103 receiver (blue curve).

**Figure 12:**
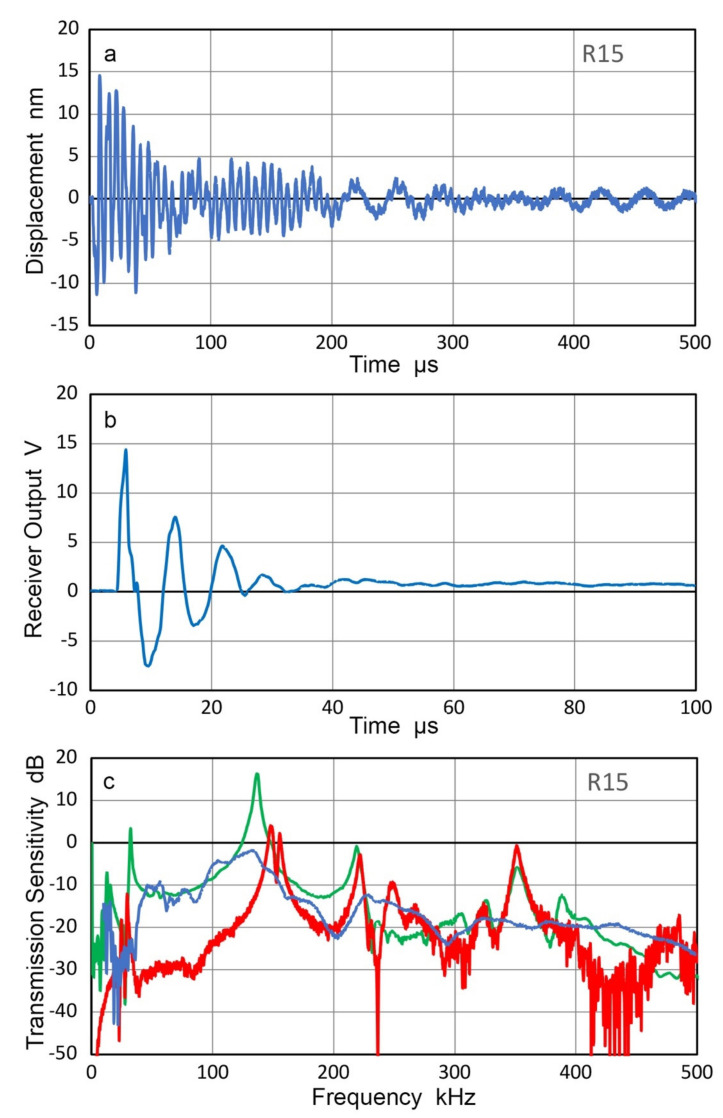
(**a**) LI-based displacement (in nm) of step-pulse excited R15 sensor. (**b**) V103 receiver output (in V) in contact with short pulse excited R15. (**c**) Transmission sensitivities of R15 from LI-based detection (red curve); from CS-16 sensor (green curve); from indirect method using V103 receiver (blue curve).

Many AE sensors utilize piezoelectric resonances in order to enhance their receiving sensitivities. When these are used as transmitters, special consideration is needed since their surface motion is affected by contacting another sensor or any medium. Transmission responses of two AE sensors, WD and R15 (Physical Acoustics, Princeton Junction, NJ, USA) were examined by laser interferometry [29]. The surface of the sensor was free. When the sensors were excited by a step pulse (385 V, 60 ns rise time), the surface motion of 25 to 30 nm peak-to-peak resulted and reverberations lasted for over 1 ms (for WD) and over 500 µs (for R15), as shown in Figure 11a and Figure 12a. When these are in contact with a receiver (Olympus V103, 1 MHz, 12.7 mm) and excited by a short pulse (373 V), much shorter receiver output of 20 to 30 µs long was produced, as illustrated in Figure 11b and Figure 12b, respectively. A Vaseline couplant was used as reported previously [21]. 

Using the receiving sensitivity of V103, T_v_ spectra were obtained for WD and R15 as transmitters and plotted by blue curves in Figure 11c and Figure 12c. The T_v_ spectra are plotted for the two sensors from laser interferometer outputs (red curves) and CS-16 outputs (green curves). As expected, LI-based and CS-based T_v_ spectra show a series of sharp resonance peaks, whereas the face-to-face experiment using V103 receiver produced damped responses. Note that the resonant frequency as a transmitter is lower than the corresponding resonant frequency as a receiver. For R15 receivers, the peak frequencies were 150–165 kHz [21,23], while the R15 transmitter (cf. Figure 12) showed peaks at 130 kHz.

These corresponded to the antiresonance and resonance frequencies of 164.4 and 136.6 kHz for R15, obtained by electrical impedance measurement using a potentiostat [21]. The terms, antiresonance and resonance, are also called parallel resonance and series resonance and refer to two resonating modes under two different (short-circuit and open-circuit) conditions, as pointed out by Uchino [30]. That is, antiresonance does not imply the absence of a resonating condition.

The results above show that the resonant AE sensors are improper choices as the source of calibration signals to accurately characterize the receiving sensitivities of other AE sensors.

### 3.5. Transmission and Receiving Sensitivities of a PZT Disk

**Figure 13:**
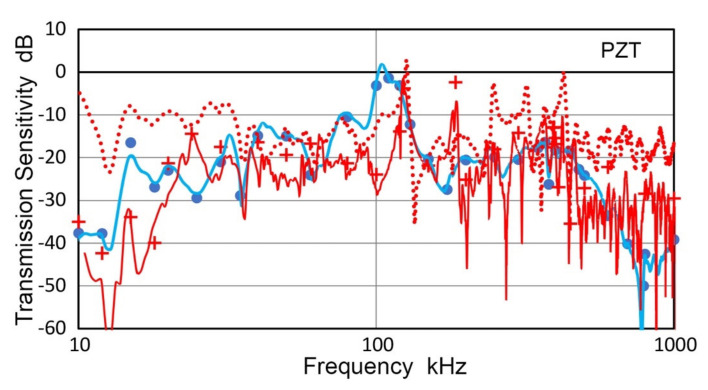
Transmission sensitivities of a PZT element by indirect method using V189 and KRNBB as receivers. Blue curve: V189, long pulse; Blue dots: V189, sine wave. Red curve: KRNBB, long pulse, back face coupled to V189; Red dot curve: KRNBB, long pulse, free back face, Red + symbols: KRNBB, sine wave.

Piezoelectric disks are the functional elements of most UT and AE transducers and their vibration behavior has been studied [21,31,32,33,34,35]. However, transmission and receiving sensitivities of a disk element are difficult to find. Using methods discussed above, a circular disk (PZT-5A ceramic, 18.0-mm diameter, 5.33-mm thickness, Valpey-Fisher, Hopkinton, MA, USA) was examined. This disk has gold coating on both faces and one face was coupled to V189 transducer. Using V189 and KRNBB as receivers, the transmission sensitivities of the PZT disk were obtained, as shown in Figure 13. Plots are in blue curve and blue dots for long pulse and sine wave excitation for V189 receiver and in red curve and red + symbols for KRNBB receiver. For the latter test, a KRNBB transducer was coupled to the free surface while the other face was on V189. This is an equivalent backing condition to that of coupling this disk to V189 transducer. Another T_v_ spectrum is also shown in Figure 13 by a red dot curve corresponding to the free vibration condition of the PZT disk. Its peak levels are 5 to 15 dB higher than those of the disk coupled to V189 on the back face as expected. The highest T_v_ values were at 104.4 kHz with V189 receiver, 101.1 kHz with V192 receiver (not shown) and at 123.5 or 126.4 kHz for KRNBB receiver, respectively. These are close to 106.3–111.1 kHz from analytical predictions for the fundamental radial resonance mode [33,34]. The difference between V189/V192 and KRNBB is a possible effect of transmitter loading since the latter has a small contact area of 1-mm diameter. Two peaks were at 390 and 421 kHz, in proximity to the nominal 400 kHz thickness resonance. The peak amplitude, however, was 10 to 15 dB lower than the amplitude of the radial resonance. When the PZT disk was free at the back face, the second highest peak was at 426.3 kHz. As the thickness is 5.33 mm and the longitudinal wave velocity is 4.323 mm/µs, the calculated thickness resonance frequency is 405.4 kHz. Thus, the observed peak frequencies were slightly higher even using a small aperture receiver. With V189 receiver, only a broad peak was present near 400 kHz. 

**Figure 14:**
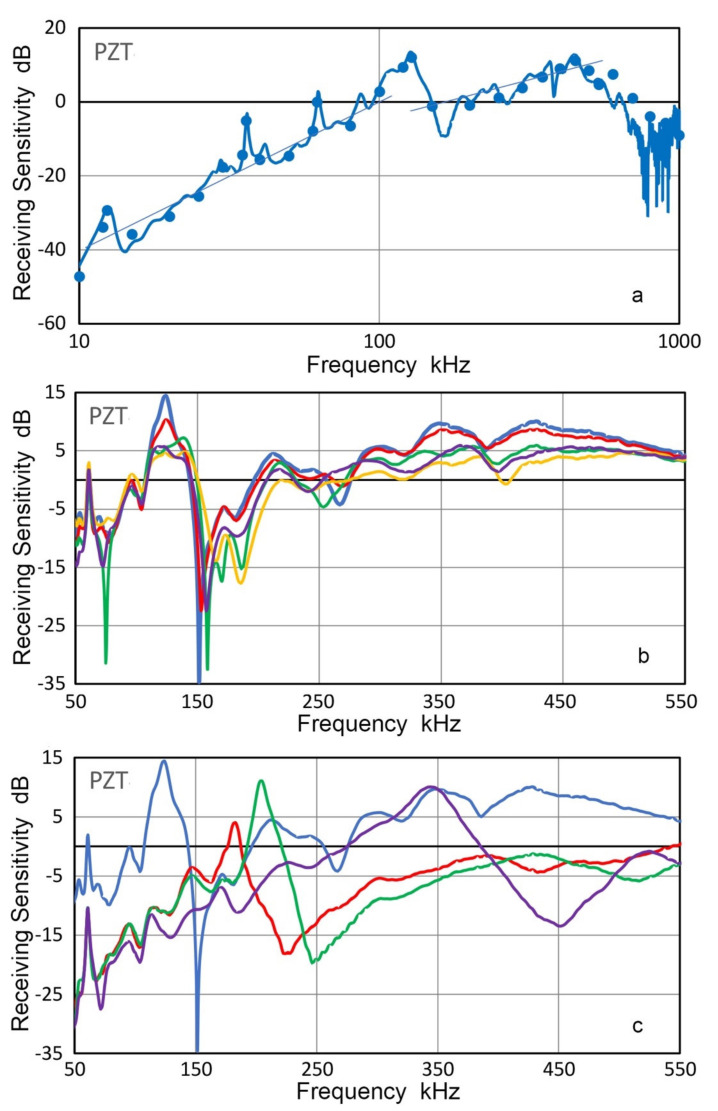
(**a**) Receiving sensitivities of 400-kHz PZT element using V189 as transmitter. Blue curve: long pulse; Blue dots: sine wave. This test used an oscilloscope probe with 10-MΩ input impedance. (**b**) Receiving sensitivities of 400-kHz PZT element with or without backing using V104 as transmitter. Blue curve: Air-backed, Red: PMMA backed (93.5-mm length, 38 × 47 mm^2^), Green: Al-backed (152-mm length, 38-mm diameter), Purple: brass-backed (90.3-mm length, 30 × 30 mm^2^), Orange: steel backed (102-mm length, 25.4-mm diameter). (**c**) Receiving sensitivities of three 1-MHz PZT disks using V104 as transmitter. Blue curve: 18-mm 400-kHz disk (same as blue curve in (**b**) above), Red: 6.3-mm PZT disk, Green: 11.5-mm PZT disk, Purple: 12.7-mm PZT disk.

Using V189 as transmitter, the receiving sensitivities of the same PZT disk were also obtained, as shown in Figure 14. Plots are in blue curve and blue dots for long pulse and sine wave excitation. Peak amplitude was at 127.3 kHz for the radial resonance and at 450 kHz for the thickness resonance. The peak frequencies were slightly higher than those of transmission sensitivities. The receiving sensitivities below these two resonances showed frequency-squared and linear frequency dependencies, as indicated by thin blue lines. These were classified as Type 2 and Type 1 frequency dependence in a previous study [36]. Type-1 behavior is for damped transducers and in the case of the PZT disk the coupling to the surface of V189 provided damping effect needed. On the other hand, the damping effect for radial motion appears to be ineffective since the PZT disk can slide over a couplant layer. 

Using another disk of 400-kHz PZT element (without lead-wires), effects of the backing on its receiving sensitivities were examined. On V104 transmitter with gold foil for grounding contact, the PZT element with gold contacts was mounted with a couplant. Its back surface was coupled to a backing material of PMMA, Al, brass and steel, as listed in Table 3. The PZT element and backing rod were pressed together under 10 to 20 N force as in face-to-face tests. For the air-backed case, a wooden rod was used to convey the force. Obtained receiving sensitivities are plotted in Figure 14b. In the case of the air-backed PZT, three main peaks were at 123.5, 348.6 and 428.2 kHz. These were slightly different from those in Figure 14a, for which the second peak was absent, but the sensitivity levels were similar. For the first peak, where radial resonance is expected, the average peak frequency was 123.5 kHz as receiver and 102.9 kHz as transmitter. This difference appears to come from the impedance maximum and minimum associated with antiresonance and resonance of the radial mode, maximizing the electrical output and displacement output, respectively. As noted in Section 3.4 above, a similar behavior was noted for resonant AE sensors, e.g., R15. 

At the main sensitivity peak (123.5 kHz in Figure 14b), metallic backing reduced receiving sensitivities by about 10 dB, whereas the reduction with PMMA was 4 dB. The sensitivity reduction, however, produced peak broadening of about 30 kHz. For the two higher frequency peaks due to the thickness resonances, effects were smaller in all the backing materials. Even though brass has the closest Z value to that of PZT, no special benefits seem to emerge from the present results. This was surprising since brass had been a favored material to avoid transmission loss. See e.g., Moffatt et al. [37]. Larger effects on the radial mode may be related to the force applied in the above tests, even though the force maximized the sensor outputs. Another test set-up is needed to avoid compressive force, but testing of disk elements with transmitters of known T_v_ spectra will be useful to design sensors for special needs. 

In addition to the 400-kHz PZT element, three other PZT-5A disks of 1-MHz thickness resonance were tested for their receiving sensitivities as well. Results are given in Figure 14c. These disks had smaller diameters of 6.3, 11.5 and 12.7 mm and showed the peak receiving sensitivities at 343.3, 204.1 and 182.2 kHz, respectively. For the two larger disks, these peaks were the highest sensitivities at frequencies below the thickness resonance peak at 1.1 MHz. The peak frequencies were 8 to 15% higher than the radial resonance frequencies (f_R_), calculated from the radial frequency constant of 2000 Hz·m for PZT-5A [32]. The observed higher peak frequencies for receiving sensitivity correspond to antiresonance frequencies (f_A_) of the radial mode since the high impedance at f_A_ contributes to improved detection. The ratio of antiresonance and resonance frequencies, f_A_/f_R_, of the radial mode is dictated by the coupling factor of piezoceramics, as discussed in [33,35]. For PZT-5A piezoceramic used here, the planar coupling factor, k_p_, for planar extension is 0.60 and Kunkel et al. [33] showed that the radial modes for such a high k_p_ value are difficult to suppress. At k_p_ = 0.60, f_A_/f_R_ is equal to 1.19 using an expression,

k_p_^2^ = 1.51 − f_R_/2f_A_ − (f_R_/f_A_)^2^ (4)

from Chen et al. [35] and is in the same range as the observed ratios of 1.08 to 1.15 for the PZT disks observed above. For R15, it was 1.15 to 1.27. Strong coupling effect for PZT-5A disks predicted by [33] implies that both f_A_ and f_R_ values should be unaffected by sensor face-loading effects. Four PZT-5A disks were subjected to transmission and receiving sensitivity tests with minimal contact to a KRNBB transducer with a small contact area. Results are summarized in Table 4. For all the disks, the receiving peak frequency was higher than the transmission peak frequency with a ratio of 1.14. This ratio can be treated as f_A_/f_R_ and its value agrees to those obtained with tightly coupling to a receiver or a transmitter. 

**Figure 15:**
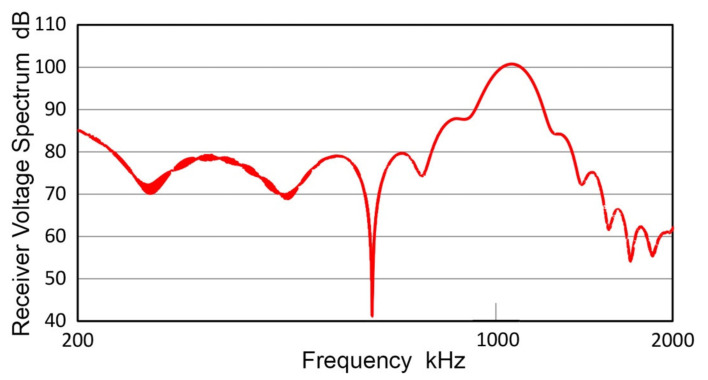
Receiver voltage spectrum of Hayward–Jackson model of an undamped 1-MHz transducer. Waveform data was read from Figure 11 of reference [38].

For a typical undamped transducer, Hayward and Jackson [38] obtained experimental and simulated waveforms for a 1-MHz transducer (given as Figure 11 in [38]) and showed more than 8–10 decaying reverberations. A step pulse excitation was used as input. When the simulated waveform is digitized and analyzed for its FFT spectrum, Figure 15 resulted, giving a narrow peak centering at 1.07 MHz with Q-value of 7.1. Frequency dependence below the peak was f^7.3^, indicating that this model had no damping term included. The model used in [38] and many others [9] had evolved from Mason’s equivalent circuit method [7], incorporating delay-line elements introduced by Redwood [8] to account for elastic wave reflections. However, it is essential to properly account for damping effects that led to sub-resonance frequency dependence as observed in receivers studied in this work and in [37]. Physically, the observed damping is a consequence of vibration absorption in the backing layer, as suggested by Redwood [8]. When wavefront reaches the back face of the sensing element, more than a half of the vibration amplitude propagates into the backing and absorbed. This energy loss process needs to be added in any simulation scheme. In these equivalent circuit models, resistive elements are expected to contribute to vibration damping, but this aspect and the energy loss process in the backing have hardly been explored. Resistive damping contributions in RCL circuits are well known, but simple RCL resonance analysis cannot be adopted except for conceptual comparison. At this stage, the governing equation of sensor dynamics derived from the damped harmonic oscillator model for inertial seismometers [39] remains to be most appropriate. See [40] for further discussion concerning its application to AE sensors and UT transducer. 

In this section, the transmission and receiving sensitivities of four PZT-5A disk elements were evaluated. In all cases, radial resonance mode was dominant at lower frequencies and thickness resonance modes suffered damping effects when a disk is coupled to a face plate or solid media. The transmission and receiving sensitivities showed peaks corresponding to the resonance and antiresonance frequencies, f_R_ and f_A_, and their ratio is governed by the coupling factor for the particular vibration mode. 

### 3.6. Receiving Sensitivities

**Figure 16:**
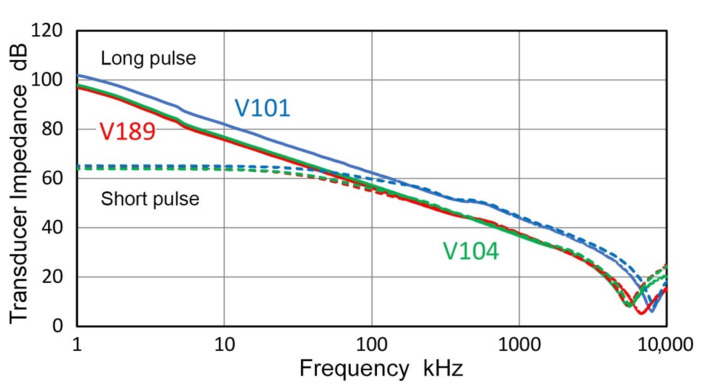
Transducer impedance of V101 (**blue curves**), V104 (**green curves**) and V189 (**red curves**) transducers. Impedance is in ohms and 0 dB corresponds to 1 Ω. Solid curves represent Z values obtained using long pulse excitation, while dash curves used short pulse excitation.

From the transmission sensitivities obtained in the preceding sections, receiving sensitivities of various transducers and sensors can be determined using the direct contact method. This procedure yielded the receiving sensitivities reported previously [21,22,23]. When the low frequency limit is reduced from 20 kHz to 1 kHz, it is necessary to consider the method of transmitter excitation. In all our recent studies of receiving sensitivities, short pulse excitation was used. That is, a pulse with a fast rise time of less than 100 ns, followed by a decay lasting less than 5 µs. It was noticed that the dynamic impedance of transmitters, which also relied on the same excitation method, started to deviate below 50–150 kHz from the expected inverse-frequency behavior due to the capacitive nature of piezoelectric transducers [22]. However, this excitation method produced consistent results in receiving sensitivities and was assumed to be adequate. As the frequency range is extended to 1 kHz, the deviation from a straight line can no longer be ignored, as shown in Figure 16 for three representative transducers, V101, V104 and V189. Solid curves represent transmitter impedance, Z, obtained by long pulse excitation using the decay time of 0.5 to 1 ms. (In these case, 10-kΩ pulser shunt resistor was used instead of usual 50 Ω.) These are almost straight below 3 MHz. Dash curves are Z values from short pulse excitation, showing a flat segment below 50 kHz, but mostly matching with the solid curves at higher frequencies. These Z values were obtained in free condition and were unaffected by transducer face loading. (Here, it should be noted that V195 transducer is an exception among the transmitters used in this study and showed a peak in Z [22]. This appears to be from an internal parallel inductor since it has a dc resistance of 0.85 Ω). 

The three transducers that exhibited smooth Z spectra in Figure 16 had jagged T_v_ spectra below approximately 100 kHz with two to four sharp dips, as shown in Figure 4. In these damped transducers, spectral irregularities in transmission output are uncorrelated to the electrical impedance. For example, V104 had three T_v_ dips at 12.4, 35.3 and 61.0 kHz (cf. Figure 4b), whereas the Z spectrum was smooth at all frequencies in Figure 16. This behavior differs from that of resonant piezoelectric transducers, of which T_v_ and R_v_ spectra showed peaks at resonance and antiresonance frequencies obtained from Z, as discussed in Section 3.5. The cause of the observed behavior of damped transducers requires a further study. 

The observed differences in Z vs. f-curves resulted from the increased frequency contents of the long pulse at low frequencies; e.g., the long pulse for V189 was 27 dB higher than the short pulse, nearly equaling the difference in Z of 28 dB at 1.9 kHz.

**Figure 17:**
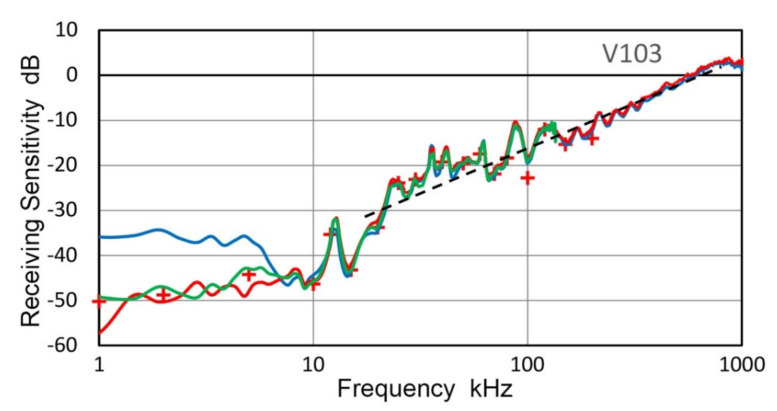
Receiving sensitivities of V103 transducer using V192 as transmitter. Blue curve: short-pulse excitation; Green curve: Gaussian-pulse excitation; Red curve: long-pulse excitation; red + symbols: sine wave excitation. Black dash line indicates linear frequency dependence.

In order to compare different excitation methods, the receiving sensitivities of V103 transducer were first examined. In addition to the short and long pulses (of ~300 V peak), a 30-kHz Gaussian pulse was utilized. The Gaussian pulse was 13.6-V peak and 22-µs length at the transmitter input and had a flat frequency response to 100 kHz, but faded above 150 kHz. Resultant receiving sensitivities of V103 are plotted in Figure 17. Effects of excitation methods are shown by color, blue: short pulse, red: long pulse, green: Gaussian pulse. Red and green curves are similar to each other, while blue curve deviated higher as frequency decreased below 10 kHz. A step pulse was also used, producing an almost identical result as the long pulse spectrum. Results of discrete sine wave excitation are given by red + symbols from 1 to 200 kHz and matched well to the red and green curves of long-pulse and Gaussian-pulse spectra. From this and other similar tests, long-pulse excitation is selected as the primary excitation method in the following tests of receiving sensitivities. Results on the receiving sensitivities of 400-kHz PZT element presented in the preceding section also used the long-pulse and sine wave excitation, giving well-matched results.

**Figure 18:**
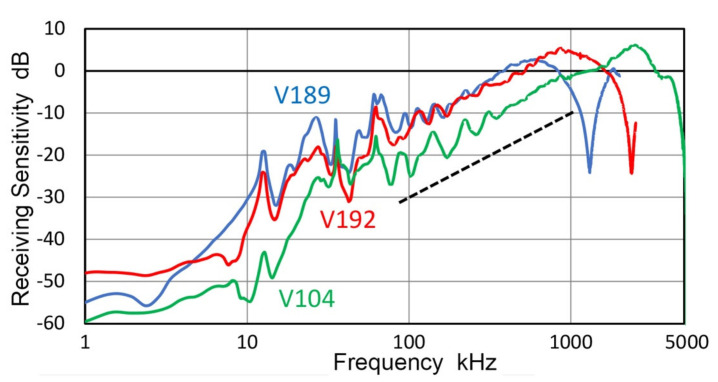
Receiving sensitivities of V104 (**green curve**), V189 (**blue**) and V192 (**red**) transducers using long-pulse excitation. Black dash line indicates linear frequency dependence.

Three representative receiving sensitivities, R_v_, of UT transducers, V104, V189 and V192, are plotted in Figure 18. These were averages of at least two R_v_ measurements with different transmitters. All three spectra and one for V103 (cf. Figure 17) exhibited linear frequency dependence below the main resonance peak and are parallel to the black dash line drawn in both figures. This is indicative of damped resonance behavior, as discussed in previous works [36,39]. This linear dependence was referred to as Type 1. In all four spectra, this Type 1 region is followed by a steep decrease (<25 kHz for V103 and V104; <12 kHz for V101 and V192), which was referred to as Type 2 with the 2nd or 3rd power of frequency. Below the Type-2 part is a region of lower frequency dependence (<10 kHz for V103 and V104; <8 kHz for V192; <3 kHz for V101). In these low frequency regions, effect of the input impedance of digital oscilloscope, Z_in_, is significant even when Z_in_ was 1 MΩ as in the present tests. Thus, the lowest frequency region is expected to show a flat response as previously observed in [36]. 

**Figure 19:**
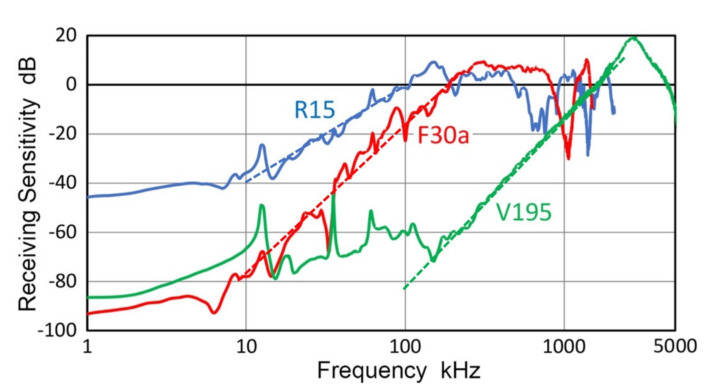
Receiving sensitivities of V195 (**green curve**), R15 (**blue**) and F30a (**red**) transducers using long-pulse excitation.

The representative receiving sensitivities, R_v_, of two AE sensors (R15 and F30a) and a UT transducer (V195) are given in Figure 19. The R15 sensor is a typical resonant sensor for general AE applications. Below its main resonance at 150 kHz, its frequency dependence followed the 2nd power of frequency and nearly flat region below 10 kHz. Frequency-squared dependence is represented by a blue dash line. F30a showed 3rd power frequency dependence below 300 kHz, down to 5 kHz, while it showed flat response over 300 to 700 kHz. Frequency-cubed dependence is represented by a red dash line. These two again showed Type-2 behavior below the main resonance. As noted earlier, V195 is a special case with an internal LC-tuning, providing a peak sensitivity at 2.8 MHz. In V195, Type-2 behavior with 3.5th-power frequency (green dash line) was seen over 150 kHz to 2 MHz with flat zone below 150 kHz.

Using improved T_v_ spectra of transmitters and long-pulse excitation method, receiving sensitivity spectra of 22 UT transducers and AE sensors were determined. Eight representative spectra are presented in this work. While the spectra for higher frequencies above 100 kHz are essentially the same as reported previously [23], the receiving sensitivity spectra in the low frequency region have become more reliable with an improved frequency resolution. Frequency dependence of R_v_ followed patterns found in a previous report [36], showing linear dependence (Type 1) in damped broadband receivers below the peak, while Type-2 behavior of 2nd or 3rd power frequency dependence was observed below the resonant peak in narrow-band sensors or the lowest peak in broadband receivers. 

### 3.7. Acoustic Impedance Mismatch 

The receiving sensitivity of an AE sensor is affected by the acoustic impedance of a structure, to which it is attached. From wave propagation theory, frequency independent displacement ratio, ξ_2_/ξ_1_, given by equation 1, dictates the sensor response [26]. However, substantial deviations were reported by the analytical and experimental studies [24,25]. Since this is a crucial element in successful conduct of AE inspection, two approaches are utilized to explore problems arising from an acoustic impedance mismatch at the sensor–structure interface. The first is to use an Al buffer plate, to which a transmitter is attached. The transmitter is excited by a pulse and the displacement on the opposite face is measured using laser interferometry. A receiver is then coupled to the buffer plate, providing its receiving sensitivity on the Al surface. This is compared to the receiving sensitivity using the face-to-face method, opposite an alumina-faced transmitter. The second approach is to use three different buffer plates, steel, Al and PMMA. This arrangement is normally used for determining the attenuation of the buffer material. Here, the attenuation and diffraction loss correction are applied in reverse to reconstruct the face-to-face result. The predicted displacement ratios are included. When the two spectral results of normal and reconstructed face-to-face test results match, the transmitting and receiving sensitivities remain the same under the buffer plate loading. From the outcome of the two approaches, it is possible to evaluate the validity of the prediction of the wave propagation theory. 

**Figure 20:**
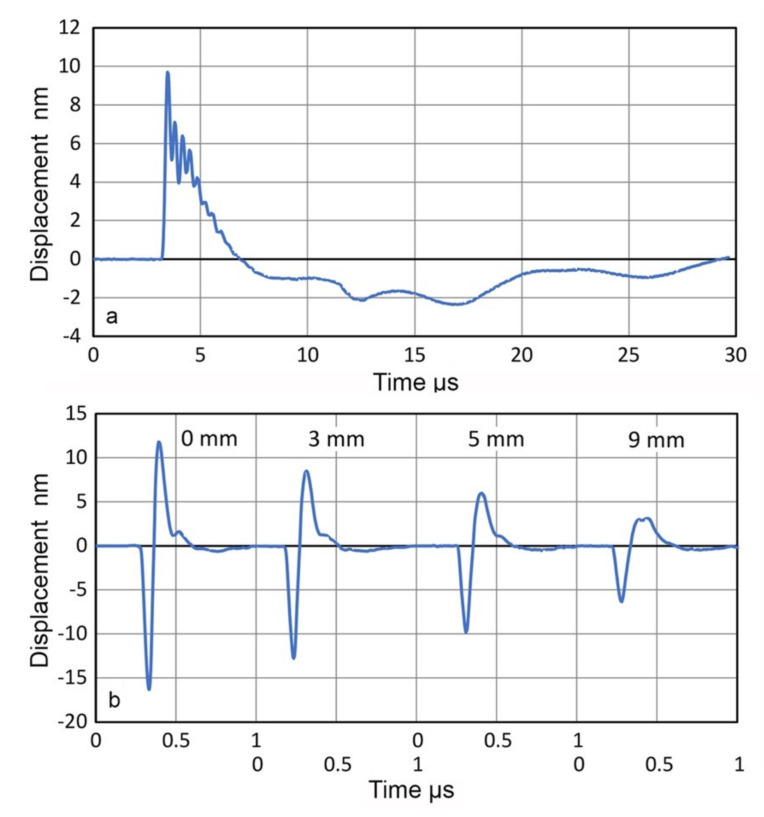
(**a**) Displacement output of FC500 transmitter. (**b**) Displacement on Al buffer plate at four positions. The distance from the center is shown above each waveform.

For the first approach, a transmitter (FC500, AET Corp, Sacramento, CA, USA) was glued to a surface of a polished buffer plate of Al7075 (38.3-mm thick, 150 × 152 mm^2^). FC500 was excited by a step pulse. The center normal displacement on free surface (before attaching to the buffer plate) was measured by using Thales SH-140 as described earlier, and is shown in Figure 20a. The peak value was 9.8 nm and it lasted over 30 µs. Displacements on the opposite face of the buffer plate were similarly measured at the center and at radial positions off center at 1, 2, 3, 4, 5, 7 and 9 mm. Four of the displacement waveforms are shown in Figure 20b. At the center (indicated as 0-mm position), the peak amplitude was −15.8 nm, which decreased at positions away from the center, down to 6.6 nm at 9-mm off. The displacements on the buffer plate were larger and much shorter, the main part lasting less than 0.5 µs (long before the reflected pulse returning after 12.2 µs). This indicates the loss of low frequency contents. The ratio of peak displacement amplitude of 1.62 = 15.8/9.8 is slightly higher than the predicted displacement ratio of 1.32 (2.41 dB), but the observed increases in displacement clearly demonstrate enlargement effects of a lower acoustic impedance medium (Z = 17.4 Mrayl for Al7075 vs. Z = 33 Mrayl for alumina). Note that sound pressure (=2πfZξ; f is frequency) is usually used in the UT literature and the sound pressure becomes lower in a low Z medium as wave enters from a high Z medium. The displacement amplitude rapidly decreased with radial distance, down to 6.6 nm at 9-mm off position. This decrease was five to ten times larger than those observed on transmitter faces, as shown in Figure 6. The large amplitude changes are expected from the thickness of the buffer plate (38.3 mm) since the near field distance at the nominal center frequency of FC500 (2.25 MHz) is 31.8 mm, which is comparable to its thickness.

**Figure 21:**
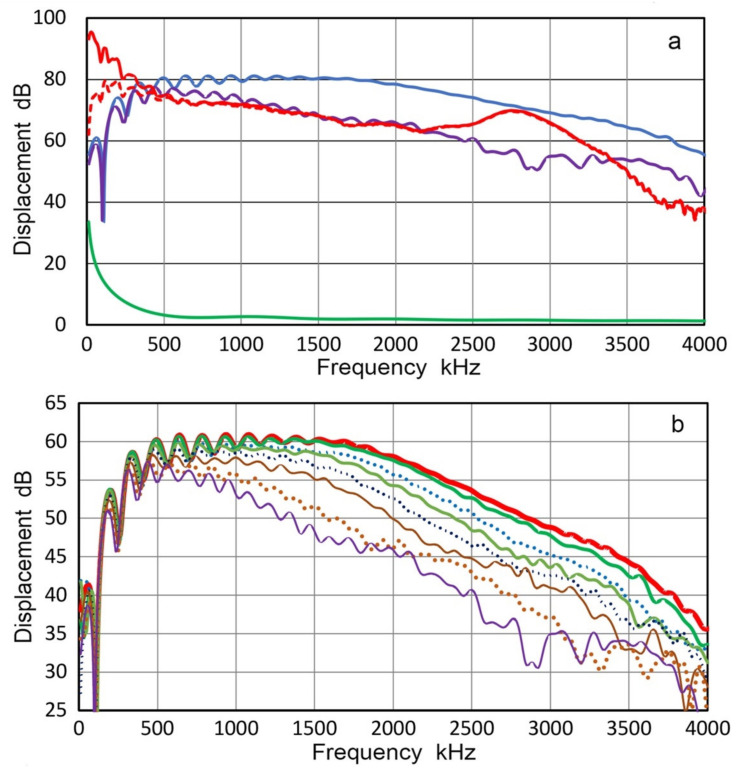
(**a**) FFT magnitude spectra of displacement waveforms. Red: On FC500 transmitter. Blue and purple: Displacement on buffer plate at the center and 9-mm off. Red dash: Corrected spectral amplitude of transmitter displacement. Green: Diffraction loss through buffer plate. (**b**) FFT magnitude spectra of displacement waveforms on buffer plate. Red: 0 mm (average of three). Green: 1 mm. Blue dot: 2 mm. Light green: 3 mm. Purple dot: 4 mm. Brown: 5 mm. Brown dot: 7 mm. Purple: 9 mm.

The FFT magnitude spectra of displacement waveforms are shown in Figure 21. In Figure 21a, red curve represents the transmitter displacement (from Figure 20a), blue and purple curves are for displacements on the buffer plate at the center and at 9-mm off. Other spectra for the displacements on the buffer plate are given in Figure 21b, including two shown in Figure 21a. The transmitter displacement spectrum was lower than that at the buffer plate center except below 300 kHz. It also showed a broad peak at 2.7 MHz, near its nominal center frequency. It appears this peak is suppressed when it is glued to the buffer plate. (Another example of face-loading effect, but this effect was absent in two other units of FC500 [21]). All the displacement spectra on the buffer plate decreased sharply with decreasing frequency below 500 kHz, where the near field distance is 4.1 mm, making the back surface well into the far field region. At higher frequencies, the radiation fields are more focused and contributed much faster decreases in spectral amplitude as the radial distance increased beyond 1 mm (green curve), as shown in Figure 21b. At 9-mm position, the amplitude loss was at 13 dB at 2 MHz. 

When waves travel within the buffer plate, viscous attenuation and diffraction effects decrease their amplitude. For the plate used, viscous attenuation coefficient, C_d_, was 10.7 dB/m/MHz [41]. This corresponds to a linear loss of 0.41 dB/MHz. Diffraction loss, D, for plate thickness x (=38.3 mm) using two circular transducers of active radius, a (=9.5 mm for FC500), is given by Rogers and van Buren [42] as

D = {[cos(2π/s) − J_1_(2π/s)]^2^ + [sin(2π/s) − J_1_(2π/s)]^2^}^0.5^ (5)

here s = x v/f a^2^, with v = 6.2 mm/µs. When these two losses are combined with the displacement ratio of +2.41 dB, no change in spectral amplitude occurred above 500 kHz, as shown by red-dash curve in Figure 21a. The red (transmitter face, center) and purple (buffer plate, 9-mm off) curves match well over 0.5 to 2.2 MHz, while the blue (buffer, center) curve was 10.4 dB on average higher than the red curve from 0.5 to 2 MHz. This is indicative of beam concentration along the beam axis in the near far-field region. 

From the above results, the use of a buffer plate of limited thickness with respect to the near field distance is undesirable for the purpose of obtaining uniform sound field. However, such a plate (or a rod) can act as a beam concentrator and has been used in transducer design.

**Figure 22:**
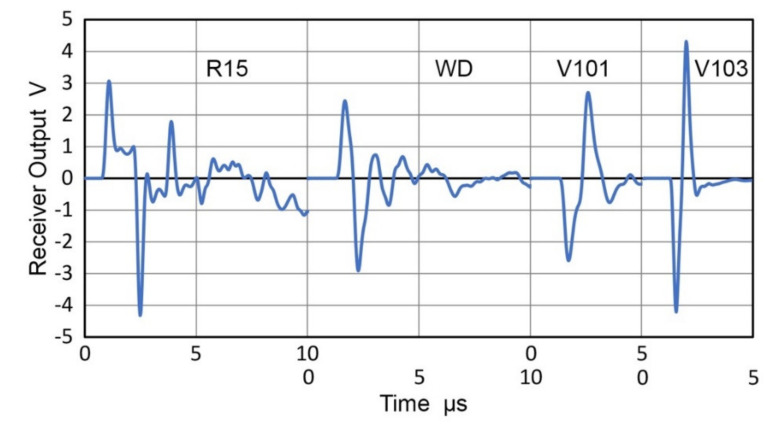
Receiver output waveforms from R15, WD, V101 and V103, coupled to the center of buffer plate. Tail parts are not shown.

**Figure 23:**
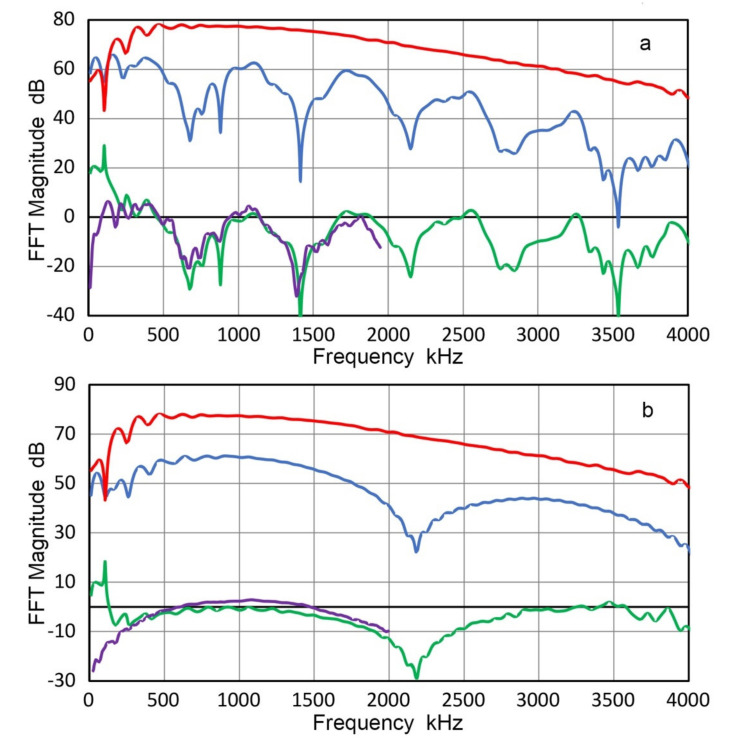
FFT magnitude spectrum of output signal of receiver on the buffer plate: (**a**) R15, (**b**) V103 (Blue curve: 0 dB reference is 1 V). The weighted average of displacement spectra of 0- to 7-mm position (Red curve: 0 dB reference is 1 nm). Receiving sensitivity (dB reference is 1 V/nm) obtained in this test (Green curve) and from a previous report (Purple curve) [23].

Two AE sensors (R15 and WD) and two UT transducers (V101 and V103) were coupled at the center of the buffer-plate back face and their output voltages were recorded, as shown in Figure 22. For these tests, FC500 transmitter was excited by the same step pulse as utilized in laser interferometry. The sensor output was bipolar, lacking the low frequency components. AE sensor outputs were longer than the first reflection (12.6 µs) and were truncated. UT transducer outputs were short and diminished before the reflection arrival. Taking the FFT magnitude spectrum of an output signal and subtracting the displacement spectrum (a weighted average of 0- to 7-mm positions or to 9-mm position for 25-mm diameter V101) from Figure 21b, the receiving sensitivity is obtained. Here, the transmission loss of 3.22 dB going from the Al buffer plate to alumina face plate of a sensor was compensated. Two examples are shown in Figure 23 for R15 and V103. In both figures, green curve is the receiving sensitivity obtained using the buffer plate. Purple curve represents the previously reported receiving sensitivity [23]. For both figures, differences are mostly below about 3 dB above 200 kHz, but can reach 10 dB or more near spectral dips, as expected. The use of displacement spectra of outside zones followed the method used in Section 3.2 because the outside zones cover larger areas and are more representative as the displacements exciting a receiving sensor. 

Because the displacements are 5 to 20 dB larger at the center or near-center zones in comparison to the outer zones, the determination of receiving sensitivities become unreliable by the use of a buffer plate unless areal displacement distributions are properly accounted for. Areal or multiple point displacement measurements are essential when a buffer plate must be utilized.

From the above results of the first approach, wave propagation theory appears to predict adequately the displacement ratio needed to account for sensor receiving sensitivity against structures of varying acoustic impedance. However, more refined areal coverages of surface displacement are required to improve the accuracy of this method.

The second approach is to compare face-to-face test results with those from the same transmitter-receiver pair but with a buffer plate between them. When the transmission loss, diffraction loss and attenuation effects are removed from the buffer-plate test result, the resultant spectrum becomes a reconstructed face-to-face result, which is equivalent to the face-to-face test result. The transmission loss, T_c_, in this test series is the product of displacement ratios, with waves going from alumina to buffer plate to alumina, and is given by [3,26]

T_c_ = (ξ_2_/ξ_1_)(ξ_3_/ξ_2_) = 4Z_1_ Z_2_/(Z_1_ + Z_2_)^2^. (6)

**Figure 24:**
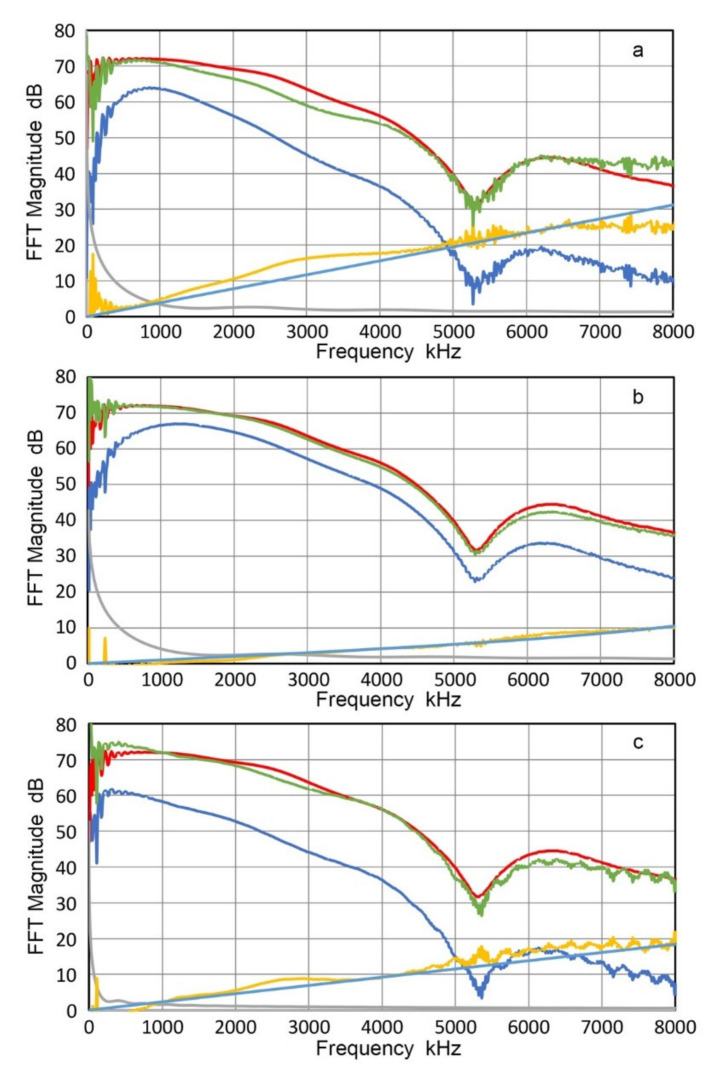
Output FFT spectra of face-to-face tests (red curves), the same for buffered tests (blue curves) and for reconstructed face-to-face tests (green curves). Transmitter was V107 and receiver was V104, both 25.4-mm diameter. Also shown by gray curves are the diffraction loss, by yellow curves observed attenuation and light blue curves fitted attenuation for viscous damping. (**a**) Steel buffer plate. (**b**) Al buffer plate. (**c**) PMMA buffer plate.

**Figure 25:**
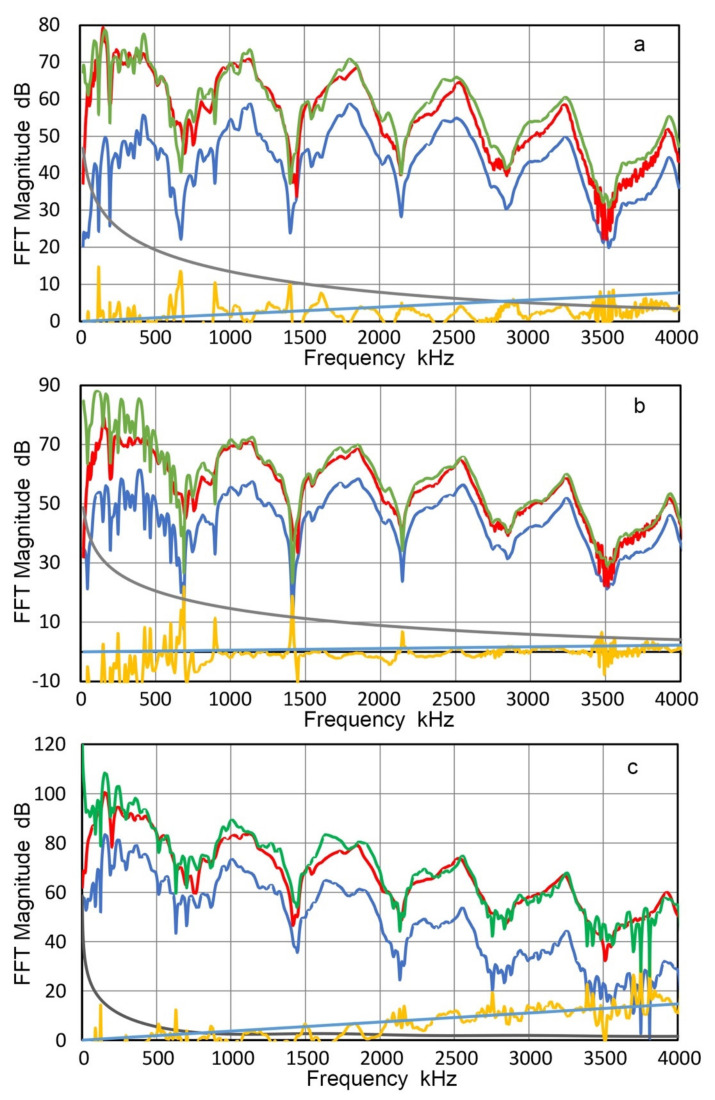
Output FFT spectra of face-to-face tests (red curves), the same for buffered tests (blue curves) and for reconstructed face-to-face tests (green curves). Transmitter was NDT C16 and receiver was R15, both 12.7-mm diameter. Also shown by gray curves are the diffraction loss, by yellow curves observed attenuation and light blue curves fitted attenuation for viscous damping. (**a**) Steel buffer plate. (**b**) Al buffer plate. (**c**) PMMA buffer plate.

Three different buffer plates were used. For steel (Z = 46.5 Mrayl), a block of 100-mm thick A533B steel plate (285 × 158 mm^2^) was used after the top and bottom surfaces were polished. This was in mill-annealed condition from Kawasaki Steel, Chiba, Japan. 110-mm long Al7075 round (Z = 17.4 Mrayl; 127-mm diameter; sample N18 in [41]) and 38.6-mm thick PMMA plate from [41] (Z = 3.20 Mrayl; 170 × 90 mm^2^) were also used. Several different transmitter-receiver pairs were used, combining 25.4-mm transducers, V101, V104 and V107 and 12.7-mm transducers and sensors, V111, NDT C16, R15 and WD. Two representative sets are given in Figure 24 for the 25.4-mm pair of V107-V104 and in Figure 25 for the 12.7-mm pair of NDT C16-R15, respectively. In both of Figure 24 and Figure 25, red curves represent the output spectra of face-to-face tests, blues curves the same for buffered tests and green curves are for the reconstructed face-to-face output spectra. The order of buffer plate material was steel, Al and PMMA for both figures, that is, Figure 24a for steel, Figure 24b for Al and Figure 24c for PMMA. The gray curves also show the diffraction loss, the yellow curves observed attenuation and light blue curves fitted attenuation for viscous damping.

When a broadband UT transducer pair is used as shown in Figure 24, the direct and reconstructed face-to-face output spectra agreed well, except for some regions (1.5 to 4 MHz and above 7 MHz for steel and under 0.5 MHz and 6 to 7 MHz for PMMA). These spectral features imply that no substantial differences exist when buffer plates with Z from 3.2 to 46 were present. When receivers were AE sensors with resonances, the general trend of good agreement between the red and green curves was again observed, except below 0.5 MHz for Al and PMMA plate cases (Figure 25b,c). Many spectral peaks and dips were observed, with peak frequencies at 1–1.2, 1.8, 2.5, 3.35, and 3.9 MHz. Thus, even when resonant sensors were included in transmitter-receiver pairs, the direct and reconstructed face-to-face output spectra agreed above 0.5 MHz and no significant effect was present on the receiving sensitivity beyond the effects of T_c_. 

**Figure 26:**
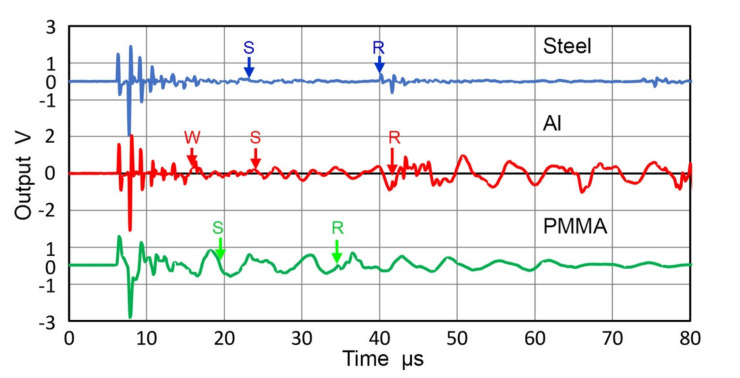
Waveforms of the receiver output signals for three different buffer plate materials, steel (**top**), Al (**middle**) and PMMA (**bottom**). Transmitter was NDT C16 and receiver was R15. Inserted letters, W, S and R, indicate arrival times corresponding to side-wall reflection, shear wave arrival and front-face reflection, respectively.

The low frequency region below 0.5 MHz in Figure 25b,c behaves differently from the same region in Figure 25a. (Here, it needs to be noted that the excitation pulse for the case of PMMA buffer plate was 10.4 dB higher in order to counter higher attenuation and the three curves (red, blue and green) are similarly higher than those in Figure 25a,b). Below 0.5 MHz, both green curves consistently exceeded the level of red curves. That is, apparent receiving sensitivity of R15 sensor was higher when it was interfaced to Al and PMMA. The increases, however, need to be ignored because the sizes of Al and PMMA buffer plates were limited and contributed extraneous effects. The effects are shown in Figure 26, which plotted R15 sensor outputs of the acoustic signals through the three buffer plates. For the Al case, side-wall reflections arrived 9.3 µs after the first pulse (marked W, on red curve in Figure 26), followed by shear wave arrival 8 µs later (marked S). These segments provided higher sensor output from resonant radial vibrations at ~130 kHz. For the PMMA case, shear wave arrival (marked S, on green curve in Figure 26) also contributed most of the low frequency oscillations. Note that the initial signals for steel and Al buffer are nearly identical, reflecting a small difference in T_c_ (0.62 dB). The initial PMMA signal level is comparable as well to the other two because a higher excitation voltage was applied as mentioned above, which compensated T_c_ difference of 9.6 dB with steel. Thus, the observed consistency in the signal levels in the three buffer materials disproves the apparent increase in R_v_. Another reason to reject the apparent R_v_ increase is the diffraction correction term. In the low frequency region, this term is large (20 to 40 dB for Al and 5 to 20 dB for PMMA), but is difficult to verify experimentally. Unless the same effect is found by using large buffer plates, it is justified to ignore the apparent increases of the receiving sensitivity of R15 from using lower acoustic impedance buffer plates of limited sizes. 

The main conclusion of this section is that the acoustic impedance mismatch effects at the sensor–structure interface can be accounted for by the transmission loss based on wave propagation theory. Two approaches used to examine this issue provided results consistent with this conclusion. 

## 4. Conclusions

This work examined the transmission characteristics of ultrasonic transducers and AE sensors for normally incident longitudinal waves since these play a critical role in ultrasonic nondestructive evaluation (NDE) methods. Both of their transmission and receiving characteristics must be known quantitatively in designing and implementing NDE applications. Even today, however, their calibration and verification have fallen behind most other aspects of NDE advances. This study first extended recent advances in quantifying the transmission and receiving sensitivities of ultrasonic transducers and acoustic emission sensors to lower frequencies using laser interferometry and an indirect method. Then, instead of relying on the central point calibration, areal and multiple sensing methods have been introduced. The benefits of multi-point sensing with an advanced laser vibrometer were demonstrated as the areas off center made dominant contribution. We also introduced the use of a small aperture receiver in conducting multi-point sensing. For areal displacement sensing, capacitive sensors were built and calibrated, relying on extant laser interferometric data. Capacitive sensors can measure area-averaged surface displacements above their low-frequency resonances. The results clearly showed the need of covering the entire area of UT transducers and AE sensors. 

In addition, we examined transducer loading effects. Combining the direct surface laser interferometry and face-to-face testing methods, changes in transmission characteristics due to front face-loading was evaluated. Damped broadband ultrasonic transducers were mostly unaffected by the loading effects. Minimal loading effects were evident from consistent T_v_ spectra found from the direct, indirect and tri-transducer methods [21,22]. 

The results showed that resonant AE sensors cannot be used as a transmitter on the basis of laser interferometry due to severe loading effects. Using refined transmission characteristics, the receiving sensitivities of transducers and sensors were reexamined, confirming frequency dependence reported previously [36]. The sensing behavior of PZT disks showed the dominance of radial resonance modes. We clarified that the peak sensitivities in transmission and reception correspond to the resonance and antiresonance of a piezoelectric resonator. AE sensors were evaluated with a buffer plate in contact with them, representing their actual usage. Results confirm that the interfacial transmission is governed by wave propagation theory, and sensor receiving sensitivities were reduced according to displacement ratios defined by acoustic impedance differences. 

## Figures and Tables

**Table 1:** Transducers used.

Transducer Model	Manufacturer	Frequency MHz	Aperture Size mm
V101	Olympus	0.5	25.4
V103	Olympus	1	12.7
V104	Olympus	2.25	25.4
V107	Olympus	5	25.4
V111	Olympus	10	12.7
V189	Olympus	0.5	38
V192	Olympus	1	38
V195	Olympus	2.25	38
F30a	Phys. Acoust.	0.3–0.7	12.7
R15	Phys. Acoust.	0.15	12.7
R15a	Phys. Acoust.	0.15	12.7
WD	Phys. Acoust.	0.1–1	12.7
FC500	AET Corp.	2.25	19
C16	NDT Systems	2.25	12.7

**Table 2:** The area fractions of contributing rings.

Area	Dia (mm)	Ring 0	Ring 1	Ring 2	Ring 3	Ring 4	Ring 5
Area 2	10.2	0.0156	0.2339	0.7505			
Area 3	15.3	0.0069	0.1040	0.3338	0.5553		
Area 4	20.3	0.0039	0.0586	0.1880	0.3127	0.4368	
Area 5	25.4	0.0025	0.0387	0.1242	0.2066	0.2885	0.3716

**Table 3:** Backing materials for PZT disk and peak heights.

Material	Air	PMMA	Al 2024	Brass C360	Steel 302SS
Z (Mrayl)	0	2.7	17.1	35.7	46.5
Length (mm)	--	93.5	152	90.3	102
Peak 1 (dB)	14.5	10.4	5.5	5.7	4.6
Peak 2 (dB)	9.7	8.5	4.3	4.2	3.0
Peak 3 (dB)	10.1	8.7	6.2	4.8	3.0

**Table 4:** Peak frequencies of PZT disks in the radial mode *.

Diameter (mm)	Transmission Sensitivity (kHz)	Receiving Sensitivity (kHz)
6.3	305.6	349.5 (343.3)
11.5	176.8	202.7 (204.1)
12.7	157.5	180.3 (182.2)
18.0	109.0 (102.9)	124.4 (125.5)

* Values in parentheses were obtained by coupling to another transducer and given in text.

## Data Availability

All the data and additional information supporting the findings of this study are available from the corresponding author upon reasonable request.
